# Dual Impact of Iron Deficiency and Antibiotics on Host Metabolism: A Tissue-Level Analysis

**DOI:** 10.3390/metabo15080549

**Published:** 2025-08-14

**Authors:** Shannon Shoff, Sydney Thomas, Peng Ji, Mariana Parenti, Carolyn M. Slupsky

**Affiliations:** 1 Department of Nutrition, UC Davis, One Shields Avenue, Davis, CA 95616, USA; shshoff@calpoly.edu (S.S.); penji@ucdavis.edu (P.J.);; 2 Department of Food Science and Technology, UC Davis, One Shields Avenue, Davis, CA 95616, USA

**Keywords:** antibiotics, hippocampus, iron deficiency, kidney, liver, metabolomics, mTOR, skeletal muscle, swine

## Abstract

**Background/Objectives**: Iron deficiency (ID) is a common nutritional deficiency in infancy and early childhood associated with increased risk of infection and increased likelihood of receiving antibiotic intervention. In the context of ID, antibiotics have been shown to exaggerate the growth impairments and negative impacts on metabolic health of ID itself. The objective of this research was to assess the tissue-level impact of antibiotics when provided during ID. **Methods**: ID was induced in piglets by withholding an iron dextran shot shortly after birth, and iron deficiency was maintained after weaning by providing an iron-deficient diet starting on postnatal day (PD) 25. Half of the ID piglets received a 3-day antibiotic course (ID + Abx) consisting of spectinomycin and gentamicin from PD34-36. The kidney, liver, skeletal muscle, and hippocampal metabolomes, as well as activity of proteins in the mTOR signaling pathway, were assessed on PD43. **Results**: While ID had minimal impacts on the liver, kidney, and skeletal muscle metabolomes, ID + Abx impaired energy metabolism and increased ketosis and oxidative stress in peripheral tissues. Hippocampal metabolites involved in neurotransmitter synthesis pathways were affected by ID and ID + Abx to a greater extent. Additionally, the activities of several proteins in the mTOR pathway were upregulated in the hippocampi of ID + Abx piglets compared to both ID and control piglets. Abx provided to iron-sufficient piglets had minimal effects on tissue metabolomes and did not alter the activity of proteins in the mTOR pathway. **Conclusions**: These results highlight that antibiotic treatment in ID alters metabolism in peripheral tissues and the developing hippocampus beyond those induced by ID alone. Considering that infants and children are develop rapidly, the combination of ID and antibiotics may have lasting impacts on neurodevelopment and cognition.

## 1. Introduction

Iron deficiency (ID) is estimated to affect 18% of children under 5 years of age worldwide [1]. ID has been associated with negative metabolic impacts that include changes in cellular energy metabolism [2,3,4], alteration of hepatic gluconeogenesis [5,6], increases in hepatic lipid content [7], and increases in lipid peroxidation in both the liver and kidney [8]. Considering that the brain develops rapidly during the first two years of life [9,10], it is not surprising that ID during this critical time has been associated with lasting deficits in learning and memory, as well as social and behavioral consequences [11,12,13]. In the brain, early-life ID has been associated with impaired energy metabolism, differences in monoamine and glutamate metabolism, an altered metabolome, as well as structural and functional abnormalities (reviewed in [14]). These changes can persist even upon normalization of brain iron content [15,16].

A dynamic interplay between the immune system and host iron status exists, such that iron deficiency impairs several aspects of cellular immunity [17,18] and may impair adaptive immunity and vaccine response [19]. Therefore, early-life ID is associated with an increased risk of morbidity [17,18] that may necessitate antibiotic treatment. Children in low- and middle-income countries (LMICs), where the greatest prevalence of ID is observed [20], have a high burden of antibiotic exposure of almost 25 antibiotic prescriptions per child by 5 years of age [21]. Although antibiotic treatment has increased survival from previously lethal infections, the impacts of antibiotics’ use extend beyond their bactericidal function. Some antibiotics are nephrotoxic and increase the risk of acute kidney injury [22,23], which may have ramifications in iron deficiency anemia (IDA), as the kidney is a key sensor of hypoxia. In the case of antibiotics that are albumin-bound, alterations in hepatic function can influence tissue distribution and clearance [22].

Swine are increasingly recognized as a valuable model for human development as they share several structural, metabolic, and neurodevelopmental similarities [24,25]. In our previous work, we observed that ID piglets had impaired growth and circulating metabolites suggestive of increased oxidative stress, ketosis, and ureagenesis, as well as altered amino acid metabolism, all of which were amplified in ID piglets concurrently treated with antibiotics [26]. Thus, the objective of this study was to examine the impacts of antibiotic use in the context of ID on the liver, kidney, and skeletal muscle, as well as the hippocampus, in the same piglets. We hypothesized that the impact of antibiotics under the conditions of ID would result in similar impacts on oxidative stress and amino acid metabolism in tissues to those observed systemically [26].

## 2. Materials and Methods

### 2.1. Piglet Housing, Diet, and Antibiotic Treatment

The animal study protocol was approved by the Institutional Animal Care and Use Committee (IACUC) of the University of California Davis (protocol numbers 20148 and 22180) and adhered to University of California Swine Facility Standard Operating Procedures.

Complete details of the animal experiment were previously published [26]. Briefly, 40 piglets of Duroc x Yorkshire x Landrace lineage were obtained from the UC Davis Swine Facility at birth. The animal study was completed in two consecutive cohorts with two litters per cohort. Piglets were sex-, weight-, and litter-matched and one sibling from each pair was randomized to Con (Cohort 1: n = 10; Cohort 2: n = 10; 5 F and 5 M per cohort) and ID (Cohort 1: n = 10; 5 F and 5 M) or ID + Abx (Cohort 2: n = 10; 4 F and 6 M). Con piglets received 200 mg of iron dextran intramuscularly on PD3, whereas ID and ID + Abx piglets received an isovolumetric injection of sterile saline. Piglets were maintained with their sow for the preweaning period. On PD25 piglets were weaned to purified control (Con: 100 mg elemental iron/kg as ferrous sulfate) or iron-deficient (ID and ID + Abx: 10 mg elemental iron/kg) diet (Envigo Teklad, Madison, WI, USA) and group-housed based on dietary treatment for the remainder of the study. Blood was collected weekly to monitor hemoglobin (Hb) concentrations and ensure piglets did not become severely anemic. Optimal status was defined at Hb ≥ 100 g/L, marginal status at 70 < Hb < 100 g/L, and anemia at Hb ≤ 70 g/L. Researchers were not blinded to treatment group.

After weaning, 3 of 10 ID + Abx piglets spontaneously developed scours necessitating antibiotic treatment. As per standard UC Davis Swine Facility protocols, all ID + Abx piglets received 50 mg spectinomycin via oral administration (SpectoGard ©, Bimeda, Irwindale, CA, USA) daily from PD34-36, as well as 5 mg gentamicin sulfate via intramuscular injection (Sparhawk Laboratories, Lenexa, KS, USA) on PD34 and PD36. The antibiotic protocol requires treatment of all piglets in the pen to prevent illness from spreading to piglets in neighboring pens. In the ID + Abx group, 3 out of 10 piglets were still exhibiting visible diarrheal symptoms on PD36 and received an additional 50 mg IM of ceftiofur on PD36 (Excede ©, Zoetis, Parsippany, NJ, USA), as per UC Davis Swine Facility protocols for treating diarrhea.

As the development of scours was spontaneous and the lingering impacts of antibiotic treatment were unexpected, a third cohort of piglets (Cohort 3: n = 12 from a single litter) was needed to assess the impact of antibiotics on otherwise healthy piglets. Piglets were maintained with their sow until PD25 and then weaned to a standard grower feed (Associated Feed & Supply Co, Turlock, CA, USA) and randomized to either Con* (n = 6) or Con* + Abx (n = 6). From PD34-36, Con* + Abx piglets received the same course of antibiotics as ID + Abx piglets.

On PD43, piglets were anesthetized with an IM injection of 0.04 mL/kg of a solution containing Telazol^TM^ (100 mg/mL), ketamine (50 mg/mL), and xylazine (50 mg/mL), then sacrificed with an overdose of pentobarbital via intracardiac injection (0.22 mL/kg, FatalPlus^TM^, Vortech Pharmaceuticals, Dearborn, MI, USA). The left lateral lobe of the liver, central cross-section of the left kidney, a portion of the left quadriceps, and the left hippocampus were collected, snap-frozen in liquid nitrogen, and stored at −80 °C until analysis.

### 2.2. Polar Metabolite Extraction

Serum metabolomics measurements were described previously [26]. All tissue samples were manually ground in liquid nitrogen using a mortar and pestle, and the resulting powder was stored at −80 °C until extraction. Polar metabolites were extracted from approximately 75 mg of muscle, kidney or liver tissue using a modified Folch extraction described by Hasegawa et al. [27]. In short, samples were extracted by first combining the tissue sample with 2:1 *v*/*v* of CHCl_3_:MeOH containing 0.002% BHT in a ratio of 12.5:1 (*w*/*v* (mg tissue/mL solvent)), followed by the addition of a buffer containing 1 mM EDTA and 0.9% KCl in a ratio of 50:1 (*w*/*v* (mg tissue/mL buffer)). The sample was then vortexed and centrifuged, and the polar layer collected. For hippocampal samples, the same method was used for extraction, with an additional extraction step using a 10:1 *v*/*v* solution of CHCl_3_:MeOH in a ratio of 21.4:1 (*w*/*v* (mg tissue/mL buffer)), vortexed, and the polar layer was combined with that of the first extraction. The polar layer was subsequently dried (miVac Duo Concentrator, GeneVac, Ipswich, UK) and the resulting pellet was stored at −80 °C until preparation for analysis.

### 2.3. NMR Data Acquisition and Spectral Analysis

The frozen dried pellets were thawed on ice and reconstituted using 270 μL of 100 mM phosphate buffer in D_2_O (pH 6.89 ± 0.5). To 207 μL of the reconstituted polar metabolites, 23 μL of an internal standard containing 4.47 mM 3-(trimethylsilyl)-1-propanesulfonic acid-d_6_ (DSS-d_6_), 0.2% NaN_3_, and 99.8% D_2_O was added, and 180 μL was subsequently transferred to 3 mm NMR tubes and stored at 4 °C until spectral acquisition. Spectra were acquired on the same day as sample preparation.

All spectra were acquired at 25 °C using the noesypr1D pulse sequence on an Avance 600 MHz spectrometer (Bruker, Billerica, MA, USA). Each spectrum was Fourier-transformed and line-broadened, then manually phased and baseline-corrected using Processor (Chenomx NMR Suite v8.6, Chenomx, Edmonton, AB, Canada). Metabolites were identified and quantified by a single researcher in Profiler, using the concentration of the internal standard (DSS-d6) as a reference (Chenomx NMR Suite v8.6, Chenomx, Edmonton, AB, Canada). Compound annotations on a representative NMR spectrum of liver tissue are shown in Figure S1. As the compound sets identified in each of the 4 tissues were similar, an unannotated representative spectrum from each tissue is shown in Figure S2. Peak assignments for creatine, phosphocreatine, and creatinine in skeletal muscle are shown in Figure S3.

### 2.4. Hippocampal Protein Extraction and Quantification

Protein from the hippocampus was extracted using two methods. In the first, approximately 50 mg of ground hippocampal tissue was extracted using standard procedures into 1 mL of ice-cold RIPA buffer (Thermo Fisher, Waltham, MA, USA) containing a 1% protease and phosphatase inhibitor cocktail (Halt™, Thermo Fisher, Waltham, MA, USA). Since detection of some proteins of interest was weak from this extraction, we utilized protein extracted from our multi-extraction method [27]. In short, we used the extraction procedure described in Section 2.2, above, using approximately 30 mg of tissue. The cell layer was lysed in buffer RLT (Qiagen, Germantown, MD, USA) containing 2-mercaptoethanol (Sigma Aldrich, St. Louis, MO, USA) and homogenized using QIAshredder homogenizer columns (Qiagen, Hilden, Germany). Protein was extracted using the AllPrep DNA/RNA/Protein Mini Kit (Qiagen, Hilden, Germany) according to manufacturer’s instructions (Qiagen, Hilden, Germany). Protein pellets from this extraction method were reconstituted in 5% SDS. Protein concentrations were quantified in triplicate using a bovine serum albumin (BSA)-based colorimetric method (DC Protein Assay Kit II, Bio-Rad, Hercules, CA, USA; SynergyH1 Plate Reader, BioTek, Winooski, VT, USA) prior to Western blot analysis.

### 2.5. Protein Expression Using Western Blot

Western blots were performed as previously described [28] with some modifications. A total of 40 µg of protein was separated on TGX stain-free gels and transferred to a low-fluorescence PVDF membrane (Bio-Rad, Hercules, CA, USA). Gels were UV-light-activated to allow for stain-free imaging of total protein, and gels were imaged before and after transfer to confirm protein transfer. Membranes were blocked using 5% non-fat milk in 1X TBS for 1 h at room temperature. The membranes were subsequently incubated overnight at 4 °C and probed (1:1000) for the following primary targets (Cell Signaling Technology, Boston, MA, USA): AKT (#9272), p-AKT(Ser473) (#4060), p-AKT(Thr308) (#13038), AMPKα (#2603), p-AMPKα(Thr172) (#2535), mTOR (#2972), p-mTOR(Ser2448) (#5536), p70-S6K(Thr389) (#9202), and p-p70-S6K(Thr389) (#9234). Membranes were washed, incubated for 1 h in the dark with goat anti-rabbit IgG (H + L) cross-adsorbed with Alexa Fluor 680 (Thermo Fisher, Waltham, MA, USA), and detected using the appropriate fluorescent channel on a ChemiDoc MP (Bio-Rad, Hercules, CA, USA). Total protein normalization was completed using Image Lab software (v6.1.0, Bio-Rad Hercules, CA, USA). We previously noted that protein extracted using the multi-extraction method provided stronger signals for phosphorylated protein targets other than p-mTOR(Ser2448) [27]. Additionally, we observed that RIPA-extracted protein provided a similar or stronger signal for total protein targets and p-mTOR(Ser2448) [27]. Thus, protein extracted using the multi-extraction method was used to quantify the expression of p-AKT(Ser473), p-AKT(Thr308), p-AMPKα, and p-p70 S6K(Thr389) for samples from Cohorts 1 and 2, as well as all targets for samples from Cohort 3. Protein extracted in RIPA buffer was used to quantify expression of total AKT, AMPKα, mTOR, p70-S6K, and p-mTOR(Ser2448) for samples from Cohorts 1 and 2. Proteins for all samples for a given target within each cohort were extracted using the same method.

### 2.6. Statistical Analysis

All statistical analyses were completed using R (v4.5.1). As no differences in the overall metabolome or protein activity were noted between the control piglets from Cohort 1 and Cohort 2 in any tissue (Figure S4), they were combined into a single control group for statistical testing. Additionally, the 3 piglets that received the additional dose of ceftiofur were retained in these analyses as they were not observed to be outliers in PCA plots of the liver, kidney, skeletal muscle, or hippocampal metabolomes, nor did excluding them alter the conclusions in our previous work [26]. All available samples were included in the analyses presented herein, with final sample sizes of Con, n = 20 (liver, muscle, hippocampus) and n = 19 (kidney); ID, n = 10 (all tissues); and ID + Abx, n = 10 (liver, muscle, hippocampus) and n = 9 (kidney).

Activity for each protein target was calculated as the ratio of phospho-to-total protein expression and normalized to the expression in Con piglets. Metabolite concentrations are expressed in µmol/g or nmol/g wet weight, as indicated, and were log_10_-transformed prior to statistical analyses to approximate a normal distribution. Overall differences in the metabolome were visualized using Principal Components Analysis (PCA). Permutational analysis of variance (PERMANOVA; Euclidean distance and 999 permutations) was applied to assess differences in centroid location using adonis2 in the vegan package (v2.6-10), and permutational analysis of multivariate dispersions (PERMDISP) was used to test for homogeneity of variance using betadsiper in the vegan package (v2.6-10). Only the kidney metabolome between treatment groups (Con, ID, and ID + Abx) and based on anemia status (optimal, marginal, and anemic) exhibited heteroscedasticity. While PERMANOVA was still applied, the variability in the data may have contributed to some of the observed differences in the kidney metabolome, thus limiting statistical power of subsequent analyses on individual metabolites using ANOVA.

The impact of treatment (Con, ID, or ID + Abx) on metabolite differences or protein activity was assessed with ANOVA using the rstatix (v0.7.2) package and effect size was determined as generalized eta-squared (η^2^_G_) using the effsize (v1.0.0) package. Thresholds of ≥0.06 and ≥0.14 were used to demarcate moderate and large effect sizes. Tukey HSD was applied for post hoc testing when a significant main effect was noted (rstatix v0.7.2). The impact of anemia (Hb < 70 mg/mL) on kidney metabolites was assessed using Mann–Whitney U tests due to heterogeneity and a large imbalance in the number of anemic vs. non-anemic piglets. All metabolite data were false discovery rate (FDR) multiple comparisons corrected based on the number of quantified metabolites within each tissue using the Benjamini–Hochberg method.

Analyses for Cohort 3 (Con*, n = 6 (kidney, liver, muscle) and n = 5 (hippocampus); Con* + Abx, n = 6 (kidney, liver, muscle) and n = 5 (hippocampus)) were completed separately from those of the main cohorts, as there was a significant gap between when the animal portion of this cohort and the main cohorts were conducted. This was due to the unexpected long-term impact of the antibiotics in the context of ID. The impact of antibiotic treatment between Con* and Con* + Abx piglets was assessed using ANOVA (rstatix v0.7.2) and FDR correction was applied for metabolite data based on the number of quantified metabolites within each tissue, as described above. Effect size between treatment groups was calculated as Hedge’s *g* using the effsize (v0.8.1) package. Thresholds of ≥0.5 and ≥0.8 were used to define moderate and large effect sizes, respectively.

For metabolite data, statistical significance and trends were defined as FDR-corrected *p* < 0.05 and FDR-corrected *p* < 0.1, respectively. Statistical significance and trends for protein targets were defined at *p* < 0.05 and *p* < 0.1, respectively.

## 3. Results

### 3.1. Impact of ID and ID + Abx on the Liver, Muscle, Kidney, and Hippocampal Metabolomes

**Figure 1:**
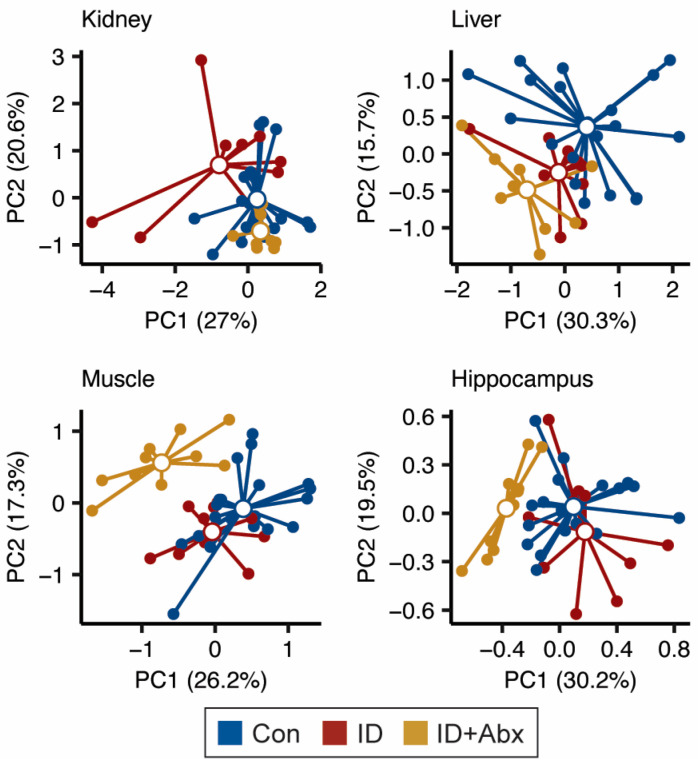
PCA plots of the kidney, liver, muscle, and hippocampal metabolomes indicate that the overall metabolism in each tissue is altered in ID (red, n = 10) and ID + Abx (yellow, n = 9–10) piglets relative to Con (blue, n = 19–20) (Pairwise PERMANOVA, Kidney: Con vs. ID: R^2^ = 0.092, *p* = 0.012; Con vs. ID + Abx: R^2^ = 0.083, *p* = 0.016; ID vs. ID + Abx: R^2^ = 0.187, *p* = 0.001; Liver: Con vs. ID: R^2^ = 0.083, *p* = 0.018; Con vs. ID + Abx: R^2^ = 0.162, *p* = 0.001; ID vs. ID + Abx: R^2^ = 0.166, *p* = 0.001; Muscle: Con vs. ID: R^2^ = 0.092, *p* = 0.009; Con vs. ID + Abx: R^2^ = 0.210, *p* = 0.001; ID vs. ID + Abx: R^2^ = 0.222, *p* = 0.001; Hippocampus: Con vs. ID: R^2^ = 0.092, *p* = 0.011; Con vs. ID + Abx: R^2^ = 0.181, *p* = 0.001; ID vs. ID + Abx: R^2^ = 0.252, *p* = 0.001).

In our previous work, we observed that the overall serum metabolome of ID piglets differed from Con piglets, with ID + Abx piglets differing considerably from both ID and Con piglets [26]. Here, a similar pattern was noted in the liver, skeletal muscle, and hippocampal metabolomes of the same piglets (Figure 1); however, this pattern was not observed in the kidney. As the kidney is a key sensor for hypoxia [29], we considered the impact of anemia status and observed that the metabolomes of anemic piglets differed from those of marginal or optimal status, regardless of treatment (Figure S5A), with PC1 reflective of Hb concentration at sacrifice (Pearson correlation, R = 0.34, *p* = 0.039). All the Con piglets exhibited optimal Hb status and ID piglets had marginal Hb or overt anemia (five marginal, five anemic), whereas most ID + Abx piglets exhibited marginal Hb status (two optimal, seven marginal, one anemic).

Overall, no differences in kidney metabolite concentrations between Con and ID piglets were observed (Table S1), and only a small number of differences were noted in the liver (Table S2) and skeletal muscle (Table S3) in the context of ID. The livers of ID piglets exhibited lower ADP, as well as higher alanine and uracil, compared to the livers of the Con piglets (Table S2). Additionally, the leucine concentrations in the skeletal muscle tended to be higher in ID piglets compared to Con (Table S3).

**Figure 2:**
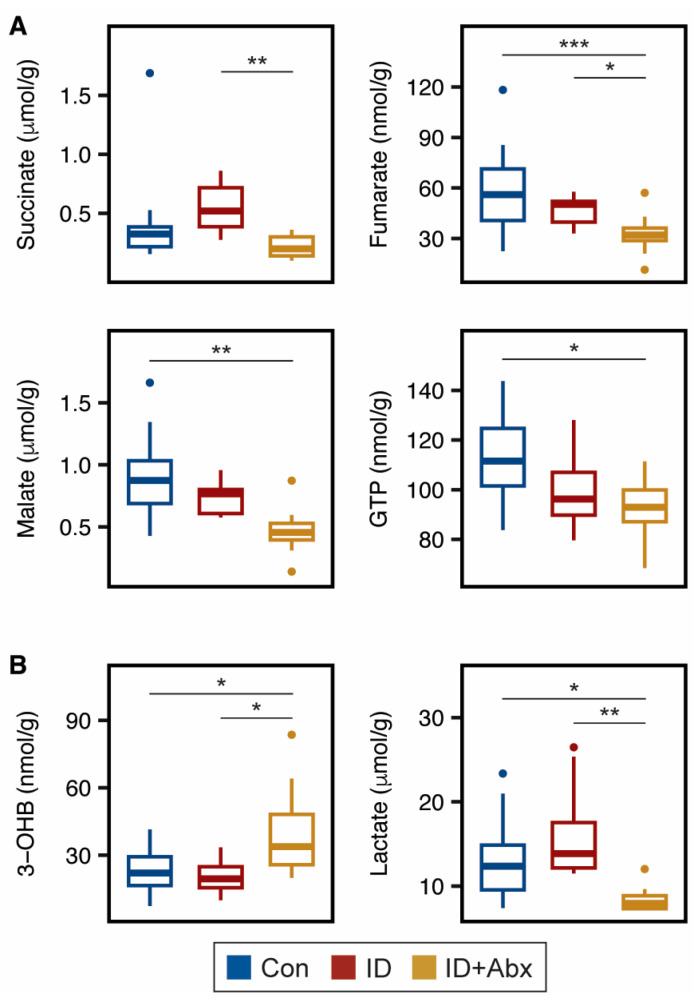
Boxplots of (**A**) TCA cycle intermediates and products in the skeletal muscle and (**B**) 3-hydroxybutyrate and lactate in the liver in ID (red, n = 10) and ID + Abx (yellow, n = 9–10) piglets relative to Con (blue, n = 19–20) (FDR-corrected ANOVA with Tukey HSD; * *p* < 0.05, ** *p* < 0.01, *** *p* < 0.001). Abbreviations: 3-OHB, 3-hydroxybutyrate.

**Figure 3:**
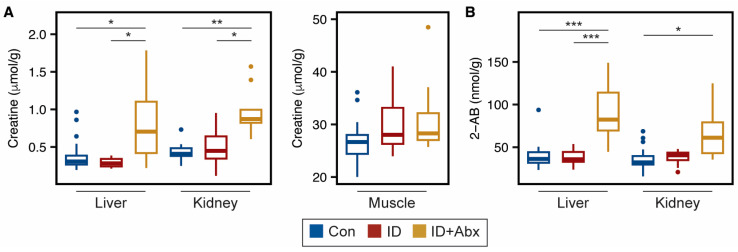
Boxplots of (**A**) creatine and (**B**) 2-aminobutyrate in liver, kidney, and muscle tissues of ID (red, n = 10) and ID + Abx (yellow, n = 9–10) piglets relative to Con (blue, n = 19–20) (FDR-corrected ANOVA with Tukey HSD; * *p* < 0.05, ** *p* < 0.01, *** *p* < 0.001). Abbreviations: 2-AB, 2-aminobutyrate.

Several energy-related metabolites were impacted by ID + Abx in the peripheral tissues. TCA cycle intermediates were impacted in skeletal muscle, with ID + Abx exhibiting lower concentrations of succinate and fumarate relative to ID piglets, and lower fumarate and malate relative to Con piglets (Figure 2A). GTP, which is a product of the TCA cycle, was also lower in the skeletal muscle of ID + Abx piglets relative to Con (Figure 2A). While hepatic TCA metabolites did not differ among treatments, ID + Abx piglets had higher concentrations of the ketone body 3-hydroxybutyrate, and lower concentrations of lactate, compared to both Con and ID piglets (Figure 2B). Furthermore, liver and kidney creatine concentrations were significantly higher in ID + Abx piglets than in ID or Con piglets (Figure 3A; Tables S1 and S2). Interestingly, no differences in creatine or phosphocreatine concentrations were noted in skeletal muscle (Figure 3B; Table S3). Endogenous creatine synthesis occurs in the liver and kidneys from the amino acids glycine, methionine, and arginine [30]. No differences were noted with glycine and methionine in these tissues (Tables S1 and S2) and arginine could not be quantified in these samples. Lastly, ID + Abx piglets also exhibited significantly higher 2-aminobutyrate in the liver compared to both Con and ID piglets and higher 2-aminobutyrate in the kidney compared to Con piglets (Figure 3B).

**Figure 4:**
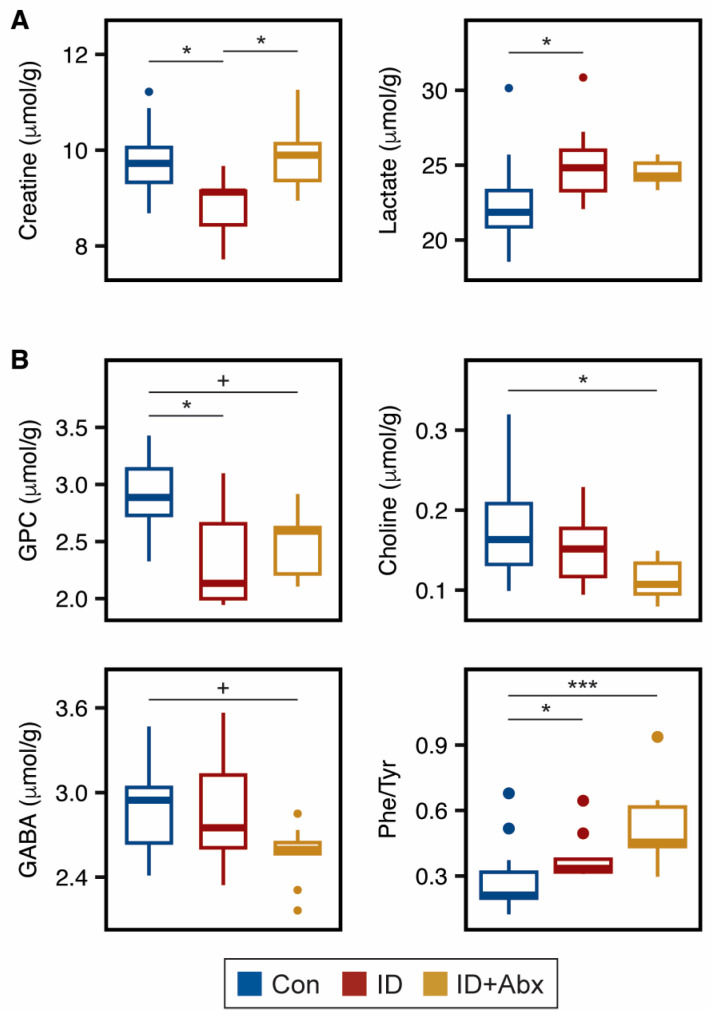
Boxplots of (**A**) energy-related and (**B**) neurotransmitter-related metabolites in the hippocampi of Con (blue, n = 20), ID (red, n = 10), and ID + Abx (yellow, n = 10) piglets (FDR-corrected ANOVA with Tukey HSD; ^+^ *p* < 0.1, * *p* < 0.05, *** *p* < 0.001). Abbreviations: GABA, γ-aminobutyric acid; GPC, glycerophosphocholine.

ID had a greater metabolic impact on the hippocampus than peripheral tissues (Table S4), with ID piglets exhibiting lower creatine and glycerophosphocholine, as well as higher lactate concentrations, compared to Con piglets (Figure 4). As with ID piglets, ID + Abx piglets also exhibited significantly lower glycerophosphocholine concentrations compared to Con, but ID + Abx piglets additionally had lower choline and tended to have lower γ-aminobutyric acid (GABA) concentrations (Figure 4B).

Phenylalanine hydroxylase metabolizes phenylalanine to tyrosine. Hippocampal phenylalanine was higher in ID compared to Con (*p* = 0.011, ES = 1.43 (95% CI: 0.58–2.29)) and tyrosine was lower in ID + Abx relative to both Con (*p* < 0.001, ES = 1.92 (95% CI: 1.00–2.48)) and ID (*p* = 0.007, ES = 1.61 (95% CI: 0.58–2.65)) (Table S4), and thus the Phe/Tyr ratio was higher in ID piglets and to a greater extent in ID + Abx piglets (Figure 4B). Interestingly, a similar pattern was observed in the serum, with ID + Abx exhibiting significantly higher Phe/Tyr than Con piglets, and ID piglets tending to differ from both the Con and ID + Abx (Figure S6). Hippocampal Phe/Tyr was strongly correlated with serum Phe/Tyr (Pearson correlation, R = 0.98, *p* < 0.001). A similar pattern in the skeletal muscle was noted, with Phe/Tyr tending to be higher in ID and significantly higher in ID + Abx compared to Con (Figure S6). Although the liver and kidney also express phenylalanine hydroxylase [31], no differences were noted in the Phe/Tyr ratio in these tissues (Figure S6).

**Figure 5:**
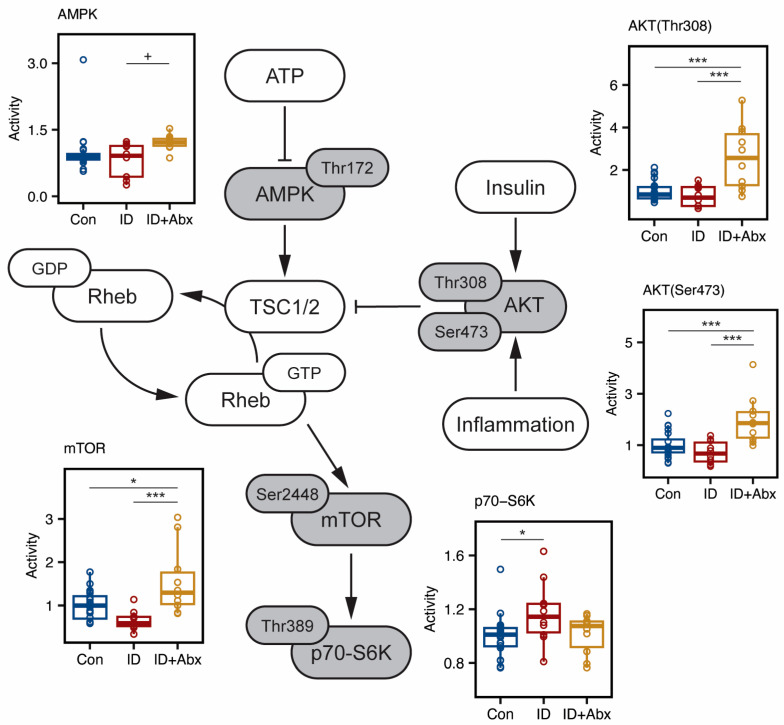
Hippocampal activity (defined as the ratio of phospho-to-total protein expression) of proteins in the mTOR pathway. Pointed arrows indicate activation and blunted ends represent inhibition of protein targets. ID piglets (red, n = 10) exhibited significantly greater S6K activity than control piglets (Con, blue, n = 20). ID + Abx piglets (yellow, n = 10) exhibited increased AKT activity at both the Thr308 and Ser473 phosphorylation sites, as well as increased mTOR activity relative to Con and ID (ANOVA with Tukey HSD, ^+^ *p* < 0.1, * *p* < 0.05, *** *p* < 0.001). Probed protein targets and phosphorylation sites are indicated in gray. Boxplots represent the median (middle line), 25th, and 75th percentiles, with whiskers indicating the lowest and highest values. Values outside of 1.5 x IQR are indicated as outliers. Dots represent values from individual piglets.

We further analyzed the activation (defined as the ratio of phospho-to-total protein expression) of several upstream regulators and downstream targets in the mTOR signaling pathway in the hippocampus (Figure S7). No differences were noted in the activation of mTOR or upstream regulators between Con and ID piglets. However, increased activation of downstream target S6K was observed in ID piglets (Figure 5). In contrast, ID + Abx piglets had significantly higher AKT activation at both the Thr308 and S473 sites, as well as elevated mTOR activity, when compared to Con and ID piglets. In addition, ID + Abx piglets tended to have increased AMPK activation; however, one Con piglet had exceptionally high AMPK activity, possibly resulting from low total AMPK expression. Statistical analysis omitting this outlier revealed significantly higher AMPK activity in ID + Abx piglets compared to both Con (*p* = 0.004) and ID piglets (*p* = 0.001).

### 3.2. Antibiotic Administration to Otherwise Healthy Piglets Has Minimal Impact

To assess the impact of antibiotics in otherwise healthy piglets, a third cohort of piglets was employed, with piglets receiving no antibiotics (Con*; n = 6) or an identical 3-day course of antibiotics administered to the ID + Abx group (Con* + Abx; n = 6), with samples collected 7 days after the last administration of antibiotics (PD43). The administration of antibiotic treatment to otherwise healthy piglets did not significantly alter the overall metabolome of the kidney, liver, or skeletal muscle (Figure S8A) 7 days after completion of antibiotic treatment, and no differences in individual metabolite concentrations were noted between Con* and Con* + Abx piglets in these tissues (kidney: Table S5; liver: Table S6; skeletal muscle: Table S7). While the overall hippocampal metabolome differed between Con* and Con* + Abx piglets (Figure S8A), the concentrations of individual metabolites did not differ (Table S8), nor did the Phe/Tyr ratio in the serum or tissues (Figure S8B). Additionally, no differences in hippocampal protein activity were observed between Con* and Con* + Abx piglets (Figure S8C).

## 4. Discussion

Several studies have shown that ID and IDA impair energy metabolism and alter nitrogen and lipid metabolism [2,5,7,32]. Interestingly, it has been reported that the magnitude of the effect seems to depend on tissue type, with liver and kidney being less affected than heart or skeletal muscle [33]. In our previous work, we reported on the lasting impacts of a 3-day antibiotic course of spectinomycin (an aminocyclitol) and gentamicin (an aminoglycoside) administered to ID piglets [26]. ID piglets who did not receive the antibiotic treatment exhibited growth faltering, elevated oxidative stress, ketosis, and alterations in amino acid metabolism. The same changes, albeit to a greater magnitude, were noted in ID + Abx piglets despite the assessment occurring 7 days after the completion of the antibiotic course. Here, we investigated the lasting tissue-level impacts of ID and antibiotic administration on liver, kidney, skeletal muscle, and hippocampal metabolism.

### 4.1. ID + Abx Negatively Influences Energy Metabolism and Oxidative Stress in Kidney, Liver, and Muscle

We observed more variability in the kidney metabolome of piglets in the ID group compared to the control and ID + Abx groups (Figure 1). A closer inspection of the data revealed that the kidney metabolome appeared to be sensitive to anemia status, as those piglets with Hb levels below the threshold for overt anemia (70 mg/mL) exhibited separation from piglets with marginal or optimal status by PCA (Figure S5). Furthermore, the kidney metabolome did not appear to be impacted by antibiotic use (Figures S5 and S8), even though gentamicin use has been associated with nephrotoxicity [34]. This suggests that the antibiotic protocol used in this study had no lasting impacts on kidney function in iron-deficient or iron-sufficient piglets. An explanation for the impact of anemia on the kidney metabolome may lie in the kidney’s critical role as a primary oxygen-sensing organ. Under hypoxic conditions, the kidney stimulates the production of erythropoietin (EPO) via a hypoxia-inducible factor (HIF)-mediated mechanism, promoting erythropoiesis in the bone marrow to restore oxygen-carrying capacity [29]. Glutathione is a natural antioxidant that mitigates oxidative stress [35], and it is regulated in part by HIF-1α. However, in the context of iron deficiency anemia, although HIF-1α may be higher in renal cells, and this is known to stimulate glutathione synthesis, perhaps glutathione is depleted more quickly to combat oxidative stress or, possibly, iron deficiency decreases the synthesis of glutathione precursors, resulting in depletion. In our study, piglets with overt anemia exhibited lower glutathione and glycine compared to non-anemic piglets (Figure S8). While these differences did not remain significant after FDR correction, the trends are consistent with glutathione depletion in the setting of anemia. This aligns with previous reports indicating that the renal metabolome is responsive to hypoxia [36]. Taken together, our findings likely reflect a hypoxia-driven metabolic shift in the kidney as a consequence of anemia.

There are indications of oxidative stress in the livers of ID + Abx piglets as well. Activation of the glutathione biosynthetic pathway in the liver as a result of oxidative stress results in increased production of the byproduct 2-aminobutyrate [37]. Elevated circulating 2-aminobutyrate and its metabolite ophthalmic acid have been suggested to be markers of hepatic glutathione depletion [37,38]. Notably, we reported that ID + Abx piglets had elevated circulating 2-aminobutyrate [26], suggestive of increased oxidative stress and an upregulation of glutathione synthesis. Interestingly, no difference in glutathione between groups was noted in the liver in this study. This may be due to the complex roles of ophthalmic acid in regulating glutathione utilization, transport, and function, as reviewed in [39]. Although ophthalmic acid could not be quantified in this study, several studies have observed differences in ophthalmic acid without corresponding alterations in glutathione levels (reviewed in [39]).

In skeletal muscle, the TCA cycle intermediates fumarate, malate, and succinate were lower in ID + Abx piglets compared to control and ID piglets, suggesting greater impairments of energy metabolism in this tissue when antibiotics are used. In line with low energy status, ID + Abx piglets exhibited increased hepatic ketone body production. The lower hepatic lactate observed may be indicative of reliance on lactate to support gluconeogenesis [40]. Additionally, creatine, which is made predominantly in the liver, kidney, and pancreas [30], was higher in the livers and kidneys of ID + Abx piglets. These results, together with the higher serum creatine previously reported in ID + Abx piglets [26], suggest that the ID + Abx piglets had increased creatine synthesis to support their systemic energy needs. Interestingly, no differences in skeletal muscle creatine were noted, even though almost all (~95%) of the body’s creatine is found in skeletal muscle [30].

### 4.2. Hippocampal Energy Metabolism Is Impacted Differently in ID and ID + Abx

ID has the potential to compromise the blood–brain barrier [41], thereby allowing aminoglycoside antibiotics (such as gentamicin) to enter the central nervous system [42]. Although minimal changes were noted in the peripheral tissues of the ID piglets, hippocampal differences between the Con and ID piglets indicated disruptions in energy metabolism, neurotransmission, and myelination, effects that were further exacerbated in the ID + Abx piglets. The elevated levels of lactate and creatine in the hippocampi of ID piglets may reflect a shift toward glycolytic metabolism, as iron deficiency is known to impair mitochondrial oxidative ATP production [3,33]. While these findings differ from previous reports suggesting that chronic hypoxia or ID do not affect hippocampal lactate levels [43,44], elevated lactate has been previously reported in the striata of ID mice [45]. Furthermore, prior studies have reported an increased phosphocreatine-to-creatine ratio in iron-deficient or hypoxic brain tissue, which may result from either reduced creatine [43,45] or elevated phosphocreatine [44]. In this study, the lower hippocampal creatine in the ID piglets may suggest an elevated phosphocreatine-to-creatine ratio, which serves as an energy reserve to support essential cellular functions. Notably, this pattern was not observed in ID + Abx piglets.

### 4.3. ID + Abx Has Greater Impact on Neurotransmitters in the Hippocampus than ID Alone

It has been reported that adolescents who are iron-deficient during infancy have increased risk of depression and anxiety [46]. Additionally, antibiotic exposure during the first two years of life has been associated with an increased risk of developing sleep disorders, ADHD, and mood and anxiety disorders between 2 and 18 years of age [47]. Thus, antibiotic use in the context of ID may further increase these risks beyond ID alone by influencing dopaminergic and GABAergic pathways. First, ID is known to impact dopaminergic signaling [48]. Endogenous dopamine biosynthesis relies on two iron-dependent hydroxylases to convert phenylalanine to tyrosine and tyrosine to L-DOPA [49]. The phenylalanine-to-tyrosine (Phe/Tyr) ratio observed in ID and, to a greater extent, in ID + Abx piglets, suggests that dopamine biosynthesis is reduced more in ID + Abx than ID alone compared to controls. Furthermore, GABAergic signaling in ID + Abx may also be impacted. While others have observed increased GABA in the hippocampi of mice who were exposed to perinatal iron deficiency [44], hippocampal GABA did not differ between Con and ID piglets in this study. This may be due to the range of anemia statuses observed in the ID piglets, as chronic hypoxia has been shown to increase hippocampal GABA [43]. In contrast, GABA tended to be lower in ID + Abx piglets. Lower GABA has been associated with a risk of depression and anxiety [50]. Interestingly, some have suggested that creatine may have neurotransmitter functions that either depress GABAergic signaling or stimulate glutamatergic signaling [51]. Thus, the changes in hippocampal creatine in ID and ID + Abx may further influence neurotransmitter signaling.

In the central nervous system, acetylcholine plays essential roles in learning and memory [52]. Both ID and ID + Abx piglets exhibited lower hippocampal glycerophosphocholine, suggesting lower acetylcholine synthesis, which could negatively impact learning and memory. Other studies using piglet models of early-life iron deficiency anemia have shown impaired spatial learning and memory [53,54]. However, choline was also noted to be lower in ID + Abx piglets, which may lead to larger impairments. Interestingly, choline supplementation has been shown to mitigate some of the memory and neurologic deficits associated with ID [55,56,57], presumably through its impact on acetylcholine synthesis. In addition, aminoglycosides can antagonize acetylcholine release, further impacting neurotransmission [58].

### 4.4. Hippocampal mTOR Signaling Is Upregulated in ID + Abx

Balanced mTOR activity is critical for neurodevelopment, as both mTOR over- and under-activation have been shown to negatively impact oligodendrocyte function and myelin structure, as well as having functional neurological consequences for learning and memory [4,59,60,61,62,63,64,65,66,67]. mTOR activity is the summation of several cellular signals, including positive regulation by insulin [68] and inflammation, and negative regulation by hypoxia and energy availability. In this study, the ID piglets exhibited elevated S6K activity, although no differences in mTOR activity or in the upstream regulators were noted. Models of non-anemic iron deficiency have shown an overall upregulation of mTOR signaling and both positive (AKT) and negative (AMPK) regulators of mTOR [67]. In contrast, models of IDA and phlebotomy-induced anemia indicate diminished mTOR signaling, partially via changes in the hypoxia-sensitive DDIT4/REDD1 pathway [69,70,71,72]. The degree of hypoxia experienced by the ID piglets in this study may have differentially influenced mTOR signaling, leading to only minimal changes in downstream targets. In contrast, the ID + Abx piglets showed marked upregulation of mTOR activity compared to both the Con and ID piglets. This pattern likely reflects the largely non-anemic status of the ID + Abx piglets, and antibiotic exposure may have further amplified mTOR-related responses.

Although the microbiome was not assessed in this study, it is reasonable to speculate that the microbiome of the ID + Abx piglets was altered via antibiotic exposure. Alterations in the gut microbiota due to antibiotic exposure can result in neuroinflammation [73]. Additionally, AKT is upregulated in the central nervous system in response to cellular stress and protects the cell through activation of key signaling pathways other than mTOR (reviewed in [74]). Taken together, these points may explain the observed upregulation of AKT activity in ID + Abx piglets. However, this response could also result in overactivation of mTOR and contribute to impairments in cognitive development.

### 4.5. Limitations and Future Directions

Disentangling the metabolic and cognitive impacts of iron deficiency, anemia-associated hypoxia, infection, and subsequent antibiotic treatment presents several challenges. Antibiotic-induced disruptions of the gut microbiome have been linked to adverse developmental outcomes during infancy [75], and alterations in the microbiota and its microbial byproducts may predispose individuals to metabolic disorders [76]. Although the microbiome was not assessed in the present study, it is well recognized that antibiotics can influence brain physiology and behavior both indirectly, through their influence on the microbiota, and directly, via host-mediated mechanisms [77]. These factors may help explain the observed differences in the hippocampal metabolomes of otherwise healthy piglets treated with a standard 3-day course of antibiotics. Future studies incorporating microbiome analyses will be essential to fully elucidate the role of gut microbial dynamics in shaping brain metabolism and function in the context of ID and antibiotics. Furthermore, the contributions of overall health status warrant additional investigation. As highlighted here and by others [43,44,45], the observed impacts of antibiotic treatment could be influenced by hypoxia resulting from anemia and, thus, warrant further investigation. Individuals with renal insufficiency are particularly susceptible to the neurotoxic effects of antibiotics [78]. Given that erythropoietin has been shown to confer neuroprotection in hypoxic-ischemic conditions [79,80,81], future research should examine whether the combined effects of iron deficiency and antibiotic exposure differ based on anemia status. Importantly, aminoglycoside use in children has been associated with an increased risk of acute kidney injury [23]. Gentamicin, which was used in this study, is frequently administered to neonates, particularly those in neonatal intensive care units (NICUs), despite its potential for inducing nephrotoxicity in these vulnerable populations [82]. Although spectinomycin is no longer prescribed for human use in the United States, it remains on the World Health Organization’s Model List of Essential Medicines and continues to be used globally [83]. As an aminocyclitol antibiotic closely related to the aminoglycoside class, spectinomycin may exert similar physiological effects.

## 5. Conclusions

In this study, we observed minimal metabolic changes in the liver, kidney, and skeletal muscle of ID piglets; however, the administration of 3-day antibiotic course of spectinomycin (an aminocyclitol) and gentamicin (an aminoglycoside) to ID piglets was associated with impaired energy metabolism, increased ketone body production, and oxidative stress in these tissues 7 days after completion of the antibiotic treatment. In the hippocampus, ID and ID + Abx impacted neurotransmitter-related metabolites differently, and mTOR signaling was upregulated in ID + Abx piglets. The results presented here and in our previous publication [26] highlight a gap in knowledge regarding long-term impacts of antibiotics in the context of nutritional deficiencies. The systemic impact of antibiotics in the context of micronutrient deficiencies may vary depending on the antibiotic and, thus, it is important to study different antibiotics and antibiotic combinations and different micronutrient deficiencies. Because iron deficiency increases susceptibility to infection, affected individuals are more likely to receive antibiotic treatment. Understanding the combined effects of iron deficiency and antibiotics, especially in rapidly developing infants and children, is critical for mitigating potential long-term consequences of concurrent exposure.

## Data Availability

The original contributions presented in this study are included in the article/Supplementary Materials. Further inquiries can be directed to the corresponding author(s).

## Supplementary Materials

The following supporting information can be downloaded at https://www.mdpi.com/article/10.3390/metabo15080549/s1. Figure S1: Annotated NMR spectrum of a representative liver sample; Figure S2: Representative NMR spectra from the liver, kidney, muscle, and hippocampus; Figure S3: NMR spectrum of skeletal muscle indicating peak assignments for creatine, phosphocreatine, and creatinine; Figure S4: PCA of kidney, liver, skeletal muscle, and hippocampal metabolomes and comparison of the activity levels of several hippocampal proteins between control piglets from Cohort 1 and Cohort 2; Figure S5: PCA and loadings of kidney metabolome by anemia status; Figure S6: Phe/Tyr ratio in serum and peripheral tissues of Con, ID, and ID + Abx piglets; Figure S7: Representative Western blot images for Con, ID, and ID + Abx piglets; Figure S8: PCA of kidney, liver, skeletal muscle, and hippocampal metabolomes, Phe/Tyr ratios and hippocampal protein expression in Con* and Con* + Abx piglets; Table S1: Kidney metabolite concentrations in Con, ID, and ID + Abx piglets; Table S2: Liver metabolite concentrations in Con, ID, and ID + Abx piglets; Table S3: Skeletal metabolite concentrations in Con, ID, and ID + Abx piglets; Table S4: Hippocampal metabolite concentrations in Con, ID, and ID + Abx piglets; Table S5: Kidney metabolite concentrations in Con* and Con* + Abx piglets; Table S6: Liver metabolite concentrations in Con* and Con* + Abx piglets; Table S7: Skeletal muscle metabolite concentrations in Con* and Con* + Abx piglets; Table S8: Hippocampal metabolite concentrations in Con* and Con* + Abx piglets.

## Abbreviations

The following abbreviations are used in this manuscript:

DSS-d6	(3-(trimethylsilyl)-1-propanesulfonic acid-d_6_
FDR	False discovery rate
GABA	γ-Aminobutyric acid
Hb	Hemoglobin
ID	Iron deficiency
LMIC	Low- and middle-income countries
PCA	Principal components analysis
PD	Postnatal day
