# Supplementary material for: Dual Impact of Iron Deficiency and Antibiotics on Host Metabolism: A Tissue-Level Analysis
Source: Metabolites. 2025 Aug 14;15(8):549. doi: 10.3390/metabo15080549 (PMC12388185; doi:10.3390/metabo15080549)
Supplement: Supplementary file 1 [file metabolites-15-00549-s001.zip › metabolites-3760864-supplementary.pdf]

Supplementary Materials

Dual Impact of Iron Deficiency and Antibiotics on Host Metabolism: A Tissue-Level Analysis

Authors: Shannon Shoff [a], Sydney Thomas [a], Peng Ji [a], Mariana Parenti [a], Carolyn M Slupsky [ab]

Table of Contents

Figures:

Figure S1. Annotated NMR spectrum of a representative liver sample.

Figure S2. Representative NMR spectra from the liver, kidney, muscle and hippocampus.

Figure S3. NMR spectrum of skeletal muscle indicating peak assignments for creatine, phosphocreatine, and creatinine.

Figure S4. PCA of kidney, liver, skeletal muscle, and hippocampal metabolomes and comparison of the activity levels of several hippocampal proteins between control piglets of Cohort 1 and Cohort 2.

Figure S5. PCA and loadings of kidney metabolome by anemia status.

Figure S6. Phe/Tyr ratio in serum and peripheral tissues of Con, ID, and ID+Abx piglets.

Figure S7. Representative Western Blot images for Con, ID, and ID+Abx piglets.

Figure S8. PCA of kidney, liver, skeletal muscle, and hippocampal metabolomes, Phe/Tyr ratios, and hippocampal protein expression in Con* and Con*+Abx piglets.

Tables:

Table S1. Kidney metabolite concentrations in Con, ID, and ID+Abx piglets.

Table S2. Liver metabolite concentrations in Con, ID, and ID+Abx piglets.

Table S3. Skeletal metabolite concentrations in Con, ID, and ID+Abx piglets.

Table S4. Hippocampal metabolite concentrations in Con, ID, and ID+Abx piglets.

Table S5. Kidney metabolite concentrations in Con* and Con*+Abx piglets.

Table S6. Liver metabolite concentrations in Con* and Con*+Abx piglets.

Table S7. Skeletal muscle metabolite concentrations in Con* and Con*+Abx piglets.

Table S8. Hippocampal metabolite concentrations in Con* and Con*+Abx piglets.

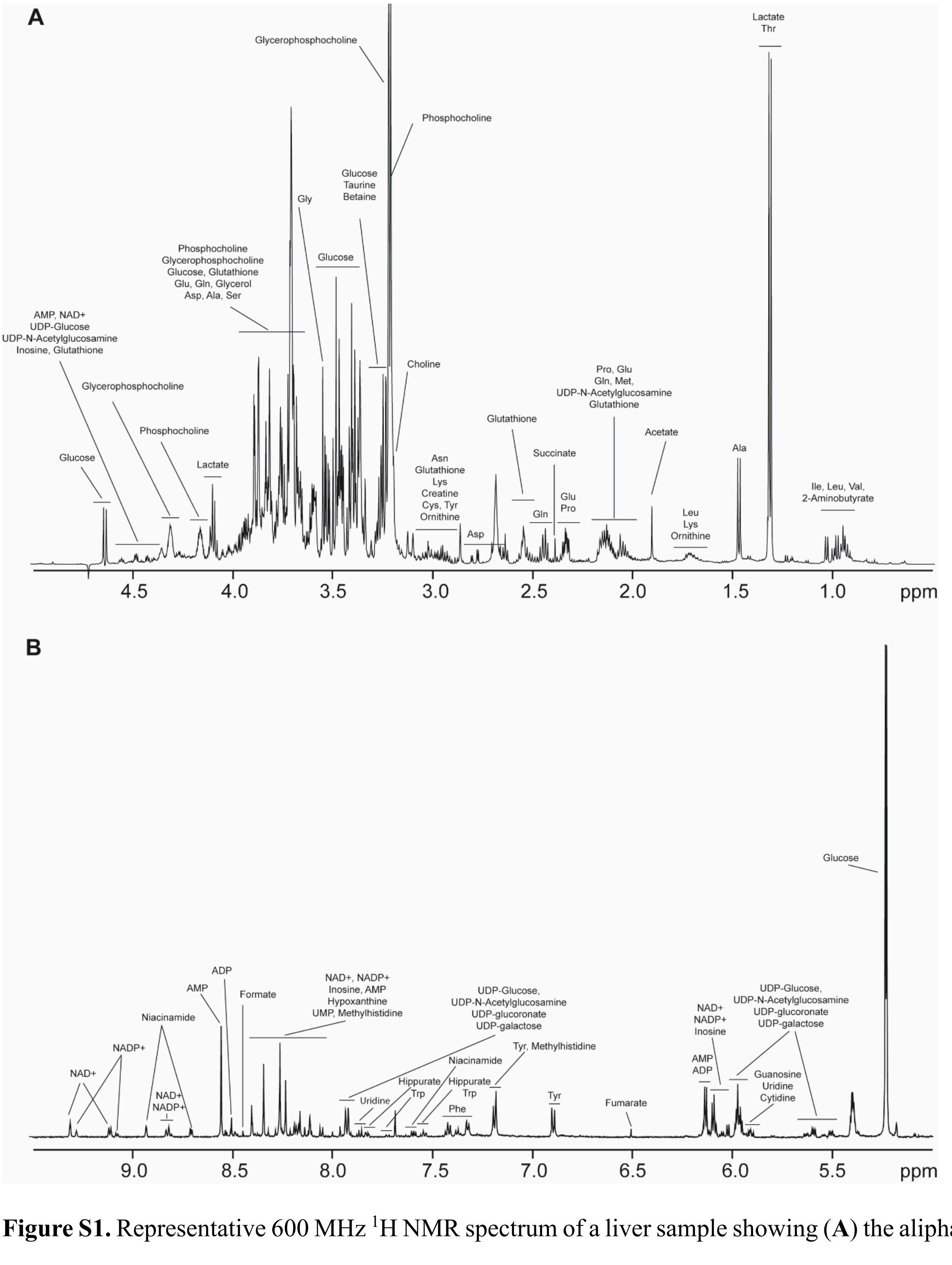

Figure S1. Representative 600 MHz ^{1}H NMR spectrum of a liver sample showing (**A**) the aliphatic and (**B**) aromatic regions of the spectrum with peak annotation. The vertical scale of the aromatic region has been increased relative to the aliphatic region for clarity.

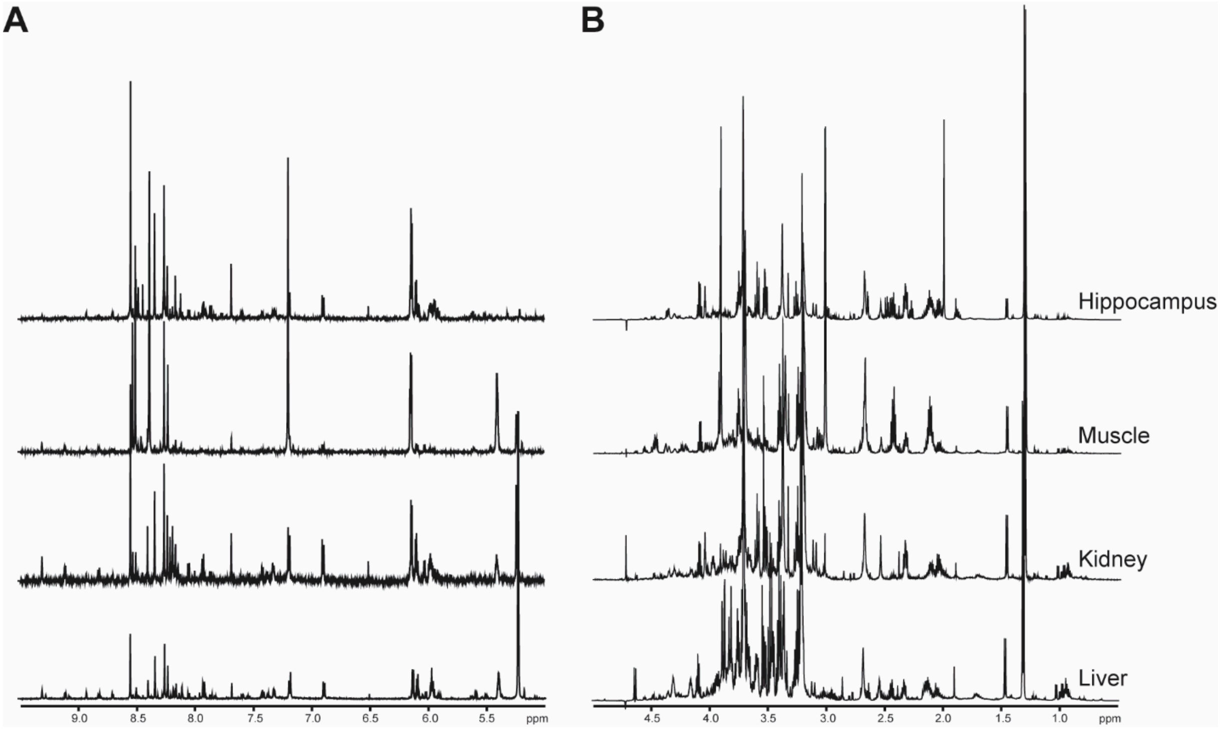

Figure S2. Representative 600 MHz ^{1}H NMR spectra from the hippocampus, skeletal muscle, kidney, and liver tissues. Spectra have been split to show regions the (**A**) aromatic and (**B**) aliphatic regions of the spectra. The vertical scale of the aromatic regions was increased relative to the aliphatic regions for clarity.

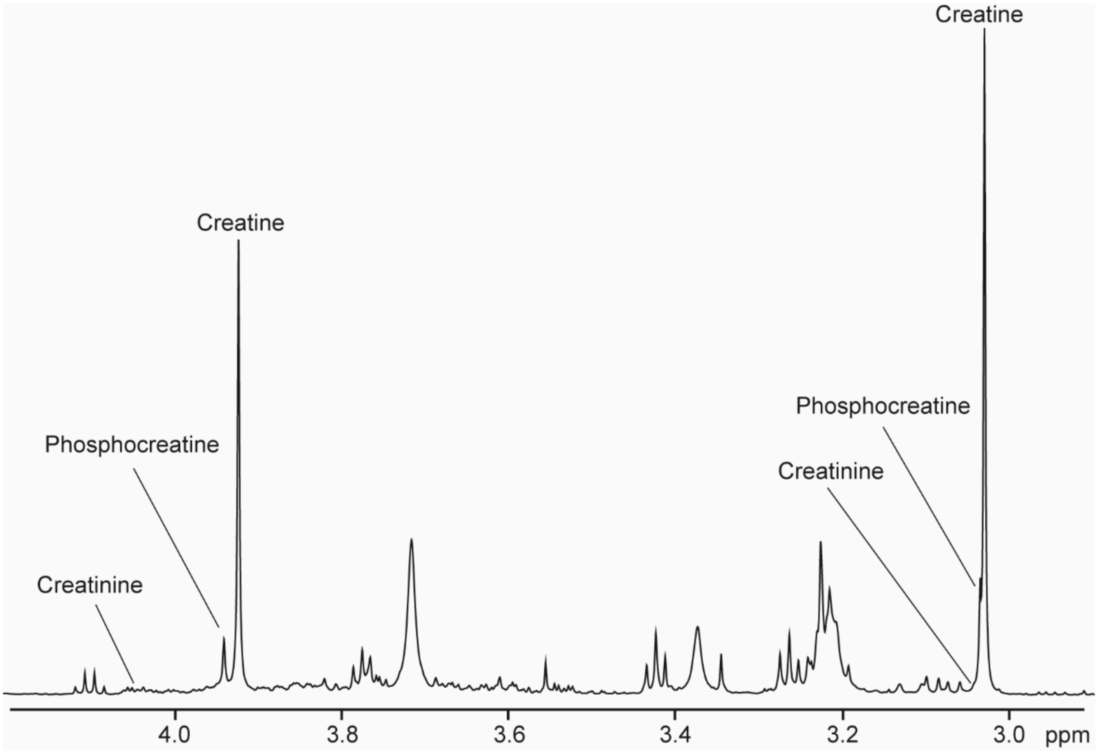

Figure S3. A 600 MHz ^{1}H NMR spectrum of skeletal muscle indicating peak assignments of creatine, phosphocreatine, and creatinine.

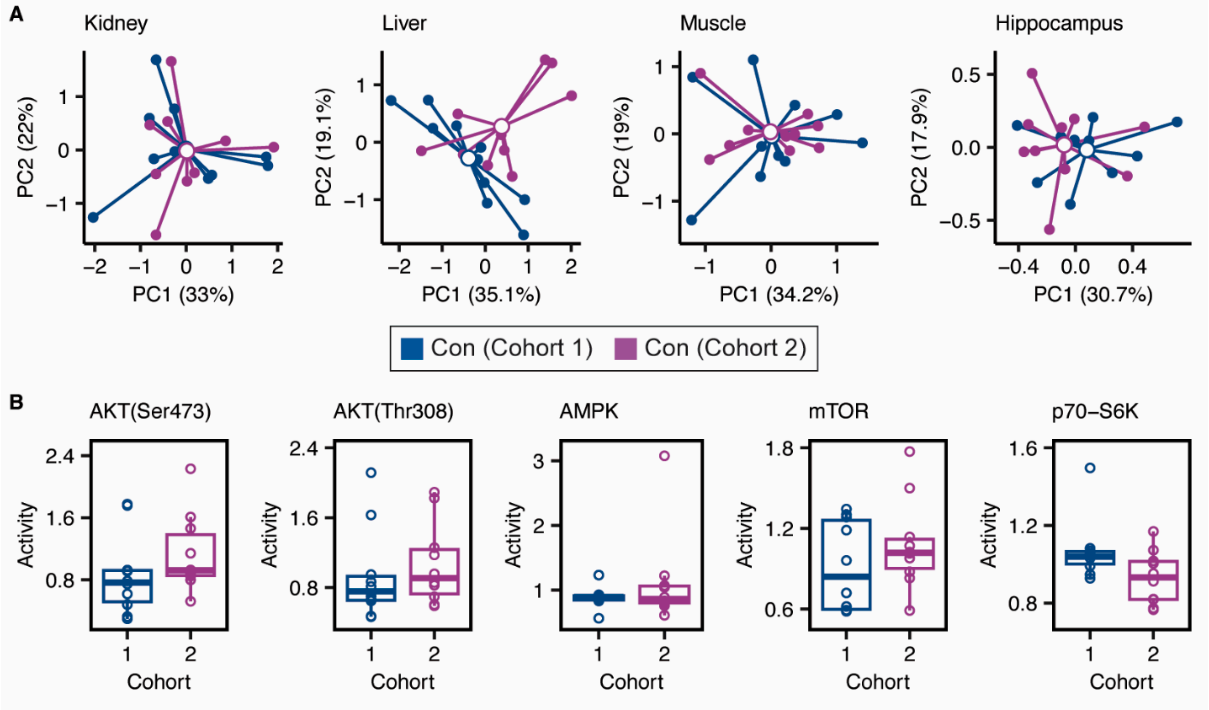

Figure S4. (**A**) PCA of the kidney, liver, muscle, and hippocampal metabolomes shows no difference in the overall metabolome between Con piglets in cohort 1 (blue, n = 10) and Con piglets in cohort 2 (purple, n = 9-10) (PERMANOVA, kidney: $R^2 = 0.058$, $p = 0.391$; liver: $R^2 = 0.089$, $p = 0.102$; muscle: $R^2 = 0.049$, $p = 0.516$; hippocampus: $R^2 = 0.058$, $p = 0.391$). (**B**) Activity (defined as the ratio of phospho-protein to total protein expression) of AKT at the Ser473 and Thr308 phosphorylation sites, AMPK, mTOR, and p70-S6K measured in the hippocampus did not differ between control piglets of Cohort 1 (blue, n = 10) and Cohort 2 (purple, n = 10) piglets (ANOVA, $p > 0.05$). Boxplots represent the median (middle line), 25th, and 75th percentiles, with whiskers indicating the lowest and highest values. Dots represent values from individual piglets.

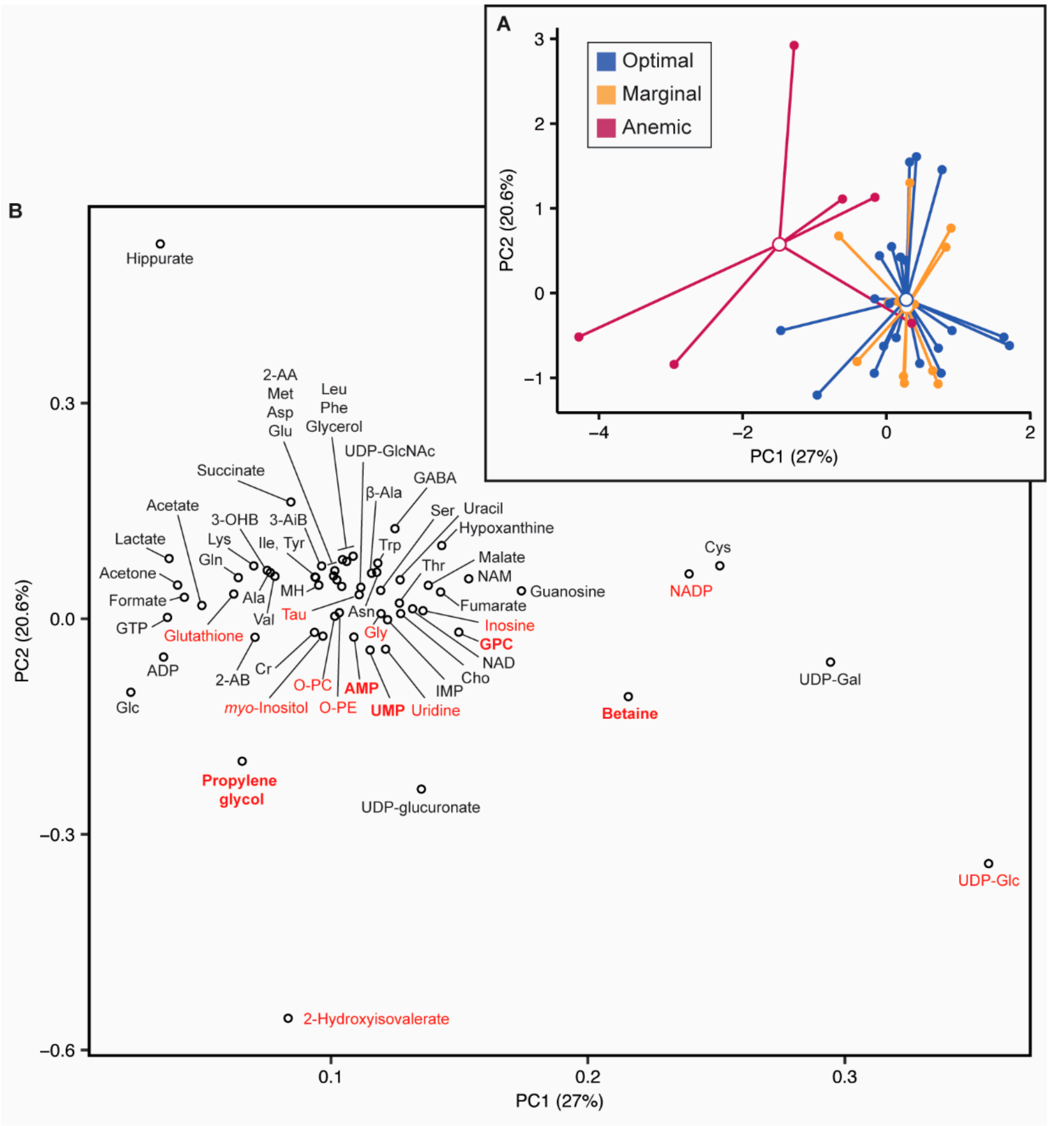

Figure S5. (**A**) PCA of the kidney metabolome strong separation of anemic piglets (pink, n = 6, Hb < 70 mg/mL) from piglets with either marginal (yellow, n = 12, 70 mg/mL ≤ Hb <100 mg/mL) or optimal (blue, n = 20, Hb ≥ 100 mg/mL) hemoglobin status at sacrifice (Pairwise PERMANOVA, optimal vs marginal: R^2 = 0.028, p = 0.546; optimal vs anemic: R^2 = 0.147, p = 0.002; marginal vs anemic: R^2 = 0.173, p = 0.002). (**B**) Loadings plot showing relationships between kidney metabolites. Red font indicates metabolites that differed significantly between anemic (n = 6) and non-anemic (marginal + optimal, n = 32) piglets (Mann-Whitney U, $p < 0.05$). Bolded red font indicates metabolites that maintained significance after FDR-correction (FDR-

corrected $p < 0.05$). Abbreviations: 2-AA, 2-aminoadipate; 2-AB, 2-aminobutyrate; 3-AiB, 3-aminoisobutyrate; 3-OHB, 3-hydroxybutyrate; β-Ala, β-alanine; Cho, Choline; Cr, creatine; GABA, γ-aminobutyric acid; Glc, glucose; GPC, glycerophosphocholine; MH, methylhistidine; NAM, niacinamide; O-PC, O-phosphocholine; O-PE, O-phosphoethanolamine; Tau, taurine; UDP-Gal, UDP-galactose; UDP-Glc, UDP-glucose, UDP-GlcNAc, UDP-N-Acetylglucosamine.

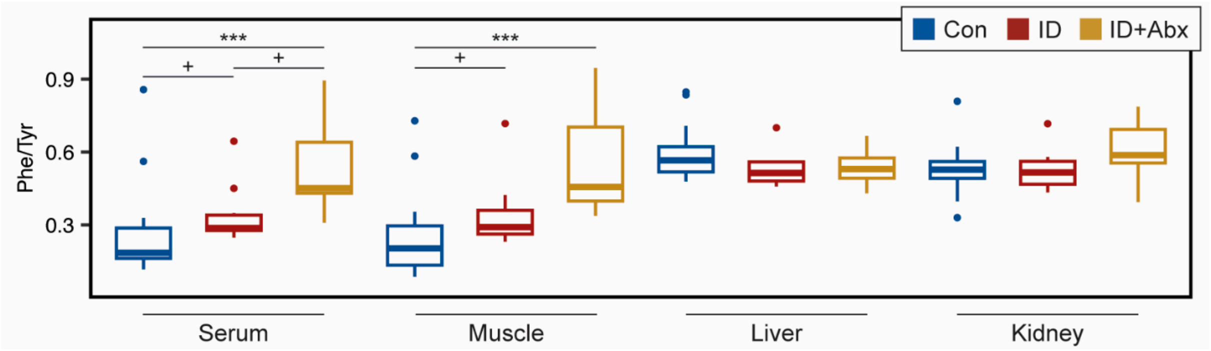

Figure S6. Boxplots of the Phe/Tyr ratio in the serum, skeletal muscle, liver, and kidney of Con (blue, n = 20 (serum, liver) and n = 19 (muscle, kidney)), ID (red, n =10), and ID+Abx piglets (yellow, n = 10 (serum, muscle, liver) and n = 9 (kidney)). Differences were assessed using ANOVA with Tukey HSD for post hoc testing. Differences between groups are noted as $^{+}$ $p < 0.1$, *** $p < 0.001$.

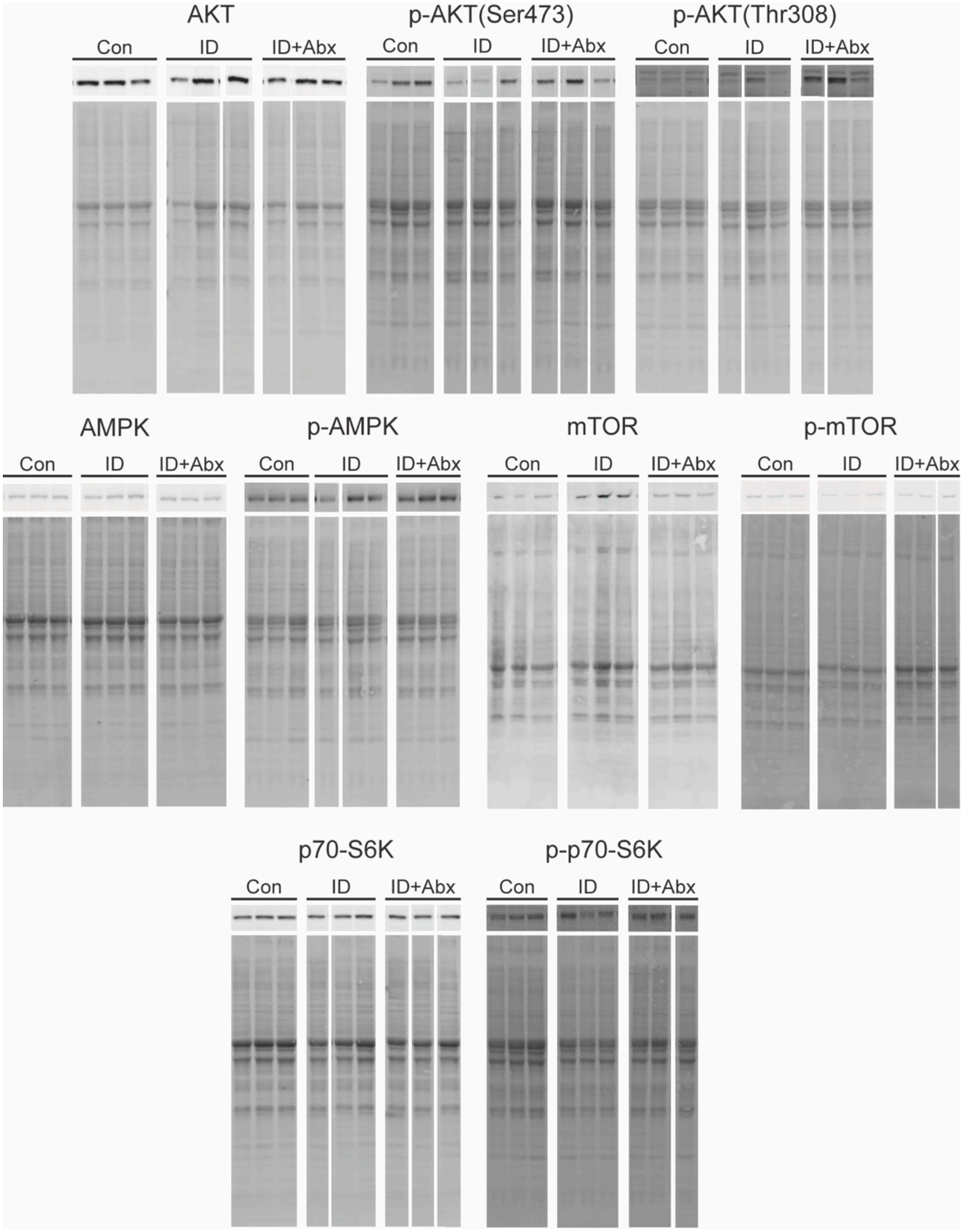

Figure S7. Representative Western Blot images from Cohort 1 and Cohort 2. Top band represents target of interest (colors have been inverted from fluorescent images for ease of visualization) with stain-free image shown below.

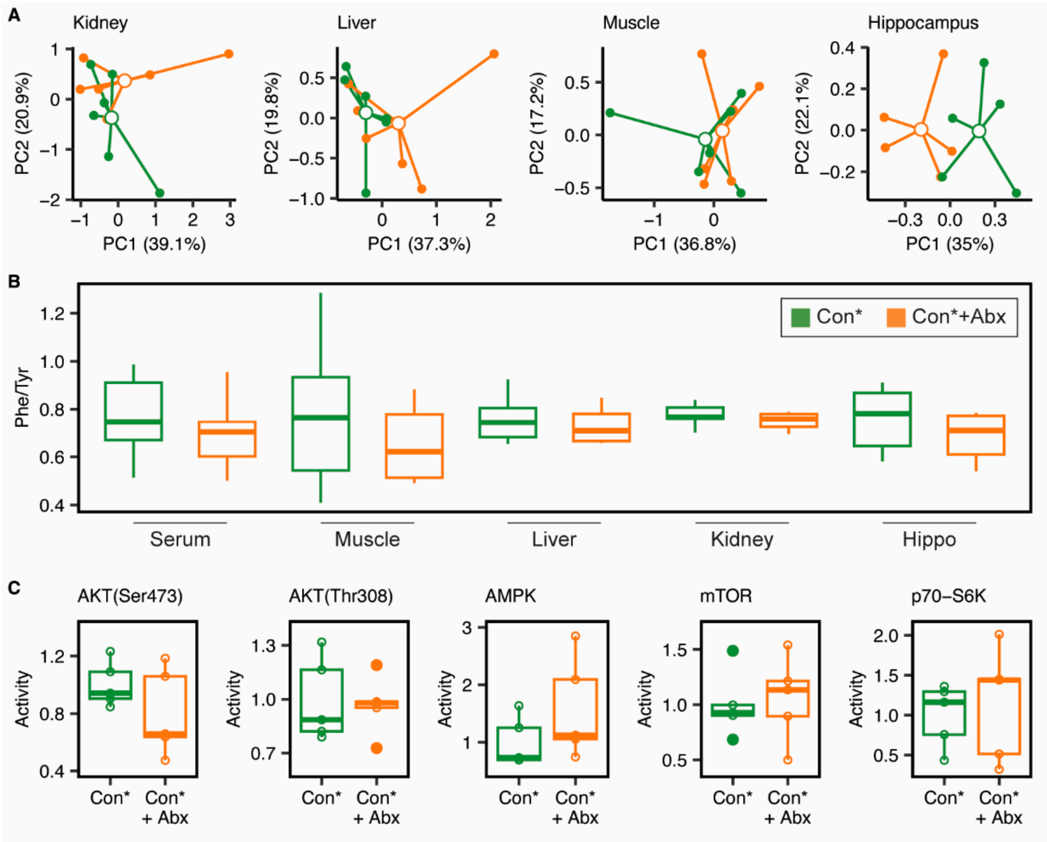

Figure S8. Comparison of control and antibiotic-treated piglets. (**A**) PCA plots indicate no difference in the overall metabolome of the kidney, liver, and muscle between control (Con*, green, n = 6) and antibiotic treated (Con*+Abx, orange, n = 6) piglets, whereas the hippocampal metabolome differed between Con* (n = 5) and Con*+Abx (n = 5) piglets (PERMANOVA, kidney: R^2 = 0.085, p = 0.491; liver: R^2 = 0.094, p = 0.385; muscle: R^2 = 0.067, p = 0.761; hippocampus: R^2 = 0.216, p = 0.031). (**B**) The Phe/Tyr ratio in serum, peripheral tissues, and hippocampus (hippo) did not differ between Con* (green, n = 6 (serum, peripheral tissues) and n = 5 (hippocampus)) and Con*+Abx piglets (orange, n = 6 (serum, peripheral tissues) and n = 5 (hippocampus)) (ANOVA, $p > 0.1$). (**C**) Activity (defined as the ratio of phospho-protein to total protein expression) of AKT at the Ser473 and Thr308 phosphorylation sites, AMPK, mTOR, and p70-S6K measured in the hippocampus did not differ between Con* (green, n = 5) and Con*+Abx

(orange, n = 5) piglets in Cohort 3 (ANOVA, $p > 0.1$). Boxplots represent the median (middle line), 25th, and 75th percentiles, with whiskers indicating the lowest and highest values. Dots represent values from individual piglets.

Table S1. Kidney metabolite concentrations in Con, ID, and ID+Abx piglets. [(1)]

Metabolite	[Con]	[ID]	[ID+Abx]	*p* [(2)]	η^2_G [(3)]
2-Aminoadipate	589 ± 354	456 ± 245	713 ± 290	0.259	0.11 (0, 0.31)
2-Aminobutyrate	37[a] ± 13	38[ab] ± 9	64[b] ± 28	**0.012**	**0.31 (0.07, 0.51)**
2-Hydroxyisovalerate	53 ± 43	22 ± 31	74 ± 35	0.124	0.19 (0, 0.4)
3-Aminoisobutyrate	32 ± 13	34 ± 14	25 ± 9	0.418	0.06 (0, 0.24)
3-Hydroxybutyrate	40 ± 17	47 ± 25	40 ± 15	0.858	0.01 (0, 0.1)
Acetate	394 ± 94	411 ± 129	429 ± 89	0.726	0.02 (0, 0.14)
Acetone	78 ± 30	96 ± 39	68 ± 21	0.423	0.06 (0, 0.23)
ADP	247 ± 56	265 ± 134	333 ± 61	0.182	0.16 (0, 0.36)
Alanine	3293 ± 885	4096 ± 1274	3367 ± 764	0.406	0.07 (0, 0.25)
AMP	1025 ± 296	833 ± 387	1196 ± 257	0.113	0.2 (0, 0.41)
Asparagine	737 ± 246	668 ± 272	536 ± 172	0.348	0.09 (0, 0.28)
Aspartate	2217 ± 696	1914 ± 680	1795 ± 653	0.348	0.09 (0, 0.28)
β-Alanine	572[a] ± 176	460[ab] ± 197	298[b] ± 44	**0.012**	**0.32 (0.07, 0.52)**
Betaine	568 ± 423	420 ± 545	600 ± 522	0.227	0.13 (0, 0.32)
Choline	921 ± 246	645 ± 312	729 ± 124	0.090	0.22 (0.01, 0.43)
Creatine	431[a] ± 103	506[a] ± 250	960[b] ± 321	**0.002**	**0.45 (0.19, 0.62)**
Cysteine	373 ± 99	312 ± 167	430 ± 103	0.259	0.11 (0, 0.31)
Formate	144 ± 35	136 ± 34	131 ± 36	0.692	0.02 (0, 0.16)
Fumarate	113 ± 26	95 ± 44	115 ± 23	0.259	0.11 (0, 0.31)
GABA	106 ± 45	123 ± 65	79 ± 13	0.537	0.04 (0, 0.2)
Glucose	2350 ± 1219	2504 ± 1383	3465 ± 928	0.155	0.17 (0, 0.38)
Glutamate	11072 ± 2353	10196 ± 3964	9629 ± 2516	0.423	0.06 (0, 0.23)
Glutamine	1410 ± 385	1730 ± 580	1143 ± 400	0.189	0.15 (0, 0.36)
Glutathione	243 ± 38	258 ± 99	294 ± 82	0.418	0.06 (0, 0.24)
Glycerol	834 ± 314	921 ± 363	690 ± 156	0.692	0.03 (0, 0.16)
Glycine	8447 ± 2496	7018 ± 2841	8387 ± 2378	0.349	0.08 (0, 0.27)
GPC	2614 ± 891	2413 ± 1673	3615 ± 1724	0.227	0.13 (0, 0.33)
GTP	85 ± 20	91 ± 29	94 ± 17	0.655	0.03 (0, 0.17)
Guanosine	99 ± 35	79 ± 41	89 ± 19	0.276	0.11 (0, 0.3)
Hippurate	11 ± 17	14 ± 18	13 ± 26	0.703	0.02 (0, 0.15)
Hypoxanthine	826 ± 287	782 ± 373	655 ± 130	0.606	0.04 (0, 0.19)
IMP	92 ± 39	72 ± 41	100 ± 21	0.174	0.16 (0, 0.37)
Inosine	446 ± 147	373 ± 176	499 ± 84	0.205	0.14 (0, 0.34)
Isoleucine	509 ± 141	481 ± 168	392 ± 37	0.348	0.09 (0, 0.27)
Lactate	12255 ± 4585	17559 ± 6432	11579 ± 2691	0.124	0.19 (0, 0.39)
Leucine	666 ± 213	683 ± 256	508 ± 58	0.418	0.06 (0, 0.24)
Lysine	774 ± 227	744 ± 209	564 ± 140	0.205	0.14 (0, 0.34)
Malate	1624 ± 488	1369 ± 616	1389 ± 243	0.349	0.08 (0, 0.27)
Methionine	336 ± 106	307 ± 113	247 ± 75	0.281	0.1 (0, 0.3)
π-Methylhistidine	323 ± 106	283 ± 99	311 ± 82	0.655	0.03 (0, 0.17)
myo-Inositol	7571 ± 1904	7047 ± 4053	10903 ± 3446	0.090	0.22 (0.01, 0.43)
NAD	373 ± 145	332 ± 154	436 ± 46	0.265	0.11 (0, 0.31)
NADP	49 ± 22	38 ± 15	53 ± 15	0.478	0.05 (0, 0.22)
Niacinamide	219 ± 66	192 ± 89	227 ± 36	0.349	0.08 (0, 0.27)
O-PC	1208 ± 383	1097 ± 440	1313 ± 153	0.358	0.08 (0, 0.26)
O-PE	2867 ± 659	2374 ± 940	3091 ± 520	0.165	0.17 (0, 0.37)
Phenylalanine	359 ± 119	349 ± 128	282 ± 55	0.514	0.05 (0, 0.21)

Metabolite	[Con]	[ID]	[ID+Abx]	p [(2)]	η^2_G [(3)]
Propylene glycol	161 ± 124	98 ± 88	218 ± 154	0.195	0.14 (0, 0.35)
Serine	1670 ± 508	1296 ± 538	1252 ± 424	0.189	0.15 (0, 0.35)
Succinate	722 ± 328	976 ± 434	434 ± 135	0.090	0.23 (0.01, 0.43)
Taurine	6505 ± 2058	4644 ± 1718	4995 ± 1299	0.105	0.21 (0.01, 0.41)
Threonine	883 ± 274	759 ± 279	861 ± 123	0.423	0.06 (0, 0.23)
Tryptophan	107 ± 37	92 ± 34	79 ± 18	0.349	0.08 (0, 0.27)
Tyrosine	686 ± 209	664 ± 253	478 ± 93	0.189	0.15 (0, 0.35)
UDP-Gal	56 ± 25	35 ± 23	56 ± 33	0.418	0.07 (0, 0.24)
UDP-Glc	77[ab] ± 42	34[a] ± 48	133[b] ± 29	**0.012**	**0.34 (0.09, 0.54)**
UDP-GlcNAc	127 ± 46	124 ± 62	130 ± 29	0.726	0.02 (0, 0.14)
UDP-glucuronate	135 ± 49	98 ± 55	138 ± 39	0.227	0.13 (0, 0.33)
UMP	130[ab] ± 31	100[a] ± 46	171[b] ± 46	**0.012**	**0.32 (0.07, 0.51)**
Uracil	83 ± 19	95 ± 47	116 ± 49	0.418	0.06 (0, 0.24)
Uridine	89 ± 32	55 ± 19	96 ± 34	0.052	0.26 (0.04, 0.47)
Valine	1034 ± 272	1044 ± 349	804 ± 55	0.291	0.1 (0, 0.29)

(1) Metabolite concentrations (mean ± SD) are expressed in nmol/g wet weight.

(2) The effect of treatment was assessed using ANOVA and FDR-correction applied based on the number of quantified metabolites in kidney tissue. Statistical significance was defined at FDR-corrected $p < 0.05$ (denoted p in the table above) and are indicated in bolded text. When a significant effect of treatment was noted, Tukey HSD with FDR-correction was used for post hoc testing. Differing superscripts indicate significant differences between treatment groups.

(3) Effect size expressed as generalized eta-squared (η^2_G) with 95% confidence interval.

Abbreviations: GABA, γ-aminobutyric acid; GPC, glycerophosphocholine; O-PC, O-phosphocholine; O-PE, O-phosphoethanolamine; UDP-Gal, UDP-galactose; UDP-Glc, UDP-glucose, UDP-GlcNAc, UDP-N-Acetylglucosamine

Table S2. Liver metabolite concentrations in Con, ID, and ID+Abx piglets. [(1)]

Metabolite	[Con]	[ID]	[ID+Abx]	p [(2)]	η^2_G [(3)]
2-Aminoadipate	359[a] ± 287	290[a] ± 157	1120[b] ± 1081	**0.005**	**0.32 (0.08, 0.51)**
2-Aminobutyrate	39[a] ± 15	38[a] ± 9	90[b] ± 35	**<0.001**	**0.57 (0.34, 0.71)**
2-Hydroxyisovalerate	15 ± 15	15 ± 14	18 ± 12	0.726	0.02 (0, 0.15)
3-Hydroxybutyrate	22[a] ± 9	20[a] ± 7	40[b] ± 21	**0.011**	**0.28 (0.05, 0.48)**
Acetate	786 ± 353	771 ± 226	833 ± 275	0.807	0.02 (0, 0.13)
Acetone	60 ± 23	51 ± 20	63 ± 22	0.502	0.05 (0, 0.22)
ADP	384[a] ± 92	233[b] ± 64	301[ab] ± 111	**0.003**	**0.35 (0.1, 0.54)**
Alanine	3606[a] ± 1248	5636[b] ± 1799	6134[b] ± 2316	**0.002**	**0.38 (0.13, 0.56)**
AMP	1430 ± 327	1092 ± 301	1173 ± 296	**0.050**[+]	0.21 (0.01, 0.41)
Asparagine	781 ± 289	767 ± 252	597 ± 129	0.196	0.12 (0, 0.31)
Aspartate	2162 ± 588	1945 ± 480	2538 ± 706	0.173	0.13 (0, 0.32)
β-Alanine	499 ± 207	708 ± 278	430 ± 111	0.050	0.21 (0.01, 0.41)
Betaine	186 ± 136	177 ± 93	181 ± 98	0.797	0.02 (0, 0.13)
Butyrate	25 ± 10	29 ± 10	56 ± 35	0.055	0.2 (0.01, 0.4)
Choline	416 ± 215	369 ± 117	551 ± 180	0.121	0.15 (0, 0.35)
Creatine	383[a] ± 214	290[a] ± 62	802[b] ± 487	**0.003**	**0.34 (0.09, 0.53)**
Cysteine	197 ± 146	335 ± 190	340 ± 130	0.126	0.15 (0, 0.34)
Cytidine	75 ± 25	82 ± 18	67 ± 15	0.378	0.07 (0, 0.25)
Formate	118 ± 45	96 ± 17	128 ± 35	0.338	0.08 (0, 0.26)
Fumarate	93 ± 35	86 ± 27	126 ± 32	**0.049**[+]	**0.22 (0.02, 0.42)**
Glucose	37289 ± 14270	52270 ± 27536	40033 ± 7255	0.196	0.12 (0, 0.31)
Glutamate	5477 ± 1371	5587 ± 1060	7006 ± 1655	0.075	0.19 (0, 0.39)
Glutamine	3949 ± 1045	4569 ± 1446	3661 ± 1108	0.364	0.08 (0, 0.26)
Glutathione	1811 ± 880	1902 ± 576	1856 ± 746	0.797	0.02 (0, 0.13)
Glycerol	1180 ± 557	1927 ± 1033	1093 ± 307	0.087	0.18 (0, 0.38)
Glycine	4809 ± 1334	4470 ± 1452	4109 ± 925	0.558	0.05 (0, 0.2)
GPC	9399 ± 4182	8721 ± 2286	8150 ± 2921	0.684	0.03 (0, 0.16)
GTP	68 ± 24	59 ± 17	61 ± 15	0.656	0.03 (0, 0.18)
Guanosine	81 ± 25	59 ± 23	76 ± 45	0.270	0.1 (0, 0.28)
Hippurate	61 ± 32	56 ± 22	73 ± 34	0.614	0.04 (0, 0.19)
Hypoxanthine	162 ± 99	149 ± 57	140 ± 73	0.849	0.01 (0, 0.11)
Inosine	696 ± 231	729 ± 265	792 ± 354	0.849	0.01 (0, 0.1)
Isoleucine	444 ± 159	396 ± 119	368 ± 89	0.391	0.07 (0, 0.24)
Lactate	13045[a] ± 4572	16189[a] ± 5555	8442[b] ± 1506	**0.002**	**0.38 (0.13, 0.56)**
Leucine	719 ± 254	694 ± 198	657 ± 130	0.849	0.01 (0, 0.1)
Lysine	700 ± 287	664 ± 212	761 ± 187	0.654	0.04 (0, 0.18)
Malate	1604 ± 508	1424 ± 413	1960 ± 518	0.126	0.15 (0, 0.34)
Mannose	691 ± 285	682 ± 293	811 ± 391	0.684	0.03 (0, 0.17)
Methionine	286 ± 111	262 ± 88	233 ± 57	0.475	0.06 (0, 0.22)
π-Methylhistidine	483 ± 128	533 ± 128	603 ± 133	0.165	0.13 (0, 0.33)
myo-Inositol	1095 ± 346	828 ± 224	1016 ± 437	0.196	0.12 (0, 0.31)
NAD	462 ± 120	391 ± 138	497 ± 175	0.389	0.07 (0, 0.25)
NADP	144 ± 39	113 ± 21	171 ± 66	0.121	0.16 (0, 0.35)
Niacinamide	399 ± 139	395 ± 174	439 ± 129	0.684	0.03 (0, 0.16)
O-PC	4229[a] ± 2083	2578[a] ± 1666	818[b] ± 316	**<0.001**	**0.59 (0.37, 0.72)**
Ornithine	378 ± 125	318 ± 106	397 ± 191	0.592	0.04 (0, 0.19)
Phenylalanine	315 ± 113	284 ± 79	250 ± 49	0.321	0.09 (0, 0.27)

**Metabolite**	**[Con]**	**[ID]**	**[ID+Abx]**	***p*** **[(2)]**	**η^2_G** **[(3)]**
Proline	677[a] ± 203	717[a] ± 156	1225[b] ± 614	**0.002**	**0.37 (0.12, 0.56)**
Propylene glycol	78 ± 107	63 ± 51	63 ± 25	0.896	0.01 (0, 0.08)
Serine	2677 ± 992	1853 ± 626	1769 ± 752	**0.029**[+]	**0.24 (0.03, 0.44)**
Succinate	399 ± 152	508 ± 206	346 ± 103	0.196	0.12 (0, 0.31)
Taurine	7676 ± 3143	5933 ± 2415	4777 ± 2650	0.115	0.16 (0, 0.36)
Threonine	873 ± 367	676 ± 202	918 ± 317	0.354	0.08 (0, 0.26)
Tryptophan	76 ± 22	75 ± 20	64 ± 17	0.391	0.07 (0, 0.24)
Tyrosine	540 ± 209	546 ± 179	473 ± 121	0.684	0.03 (0, 0.17)
UDP-Gal	236 ± 97	281 ± 90	351 ± 134	0.126	0.15 (0, 0.34)
UDP-Glc	750 ± 305	801 ± 258	1125 ± 453	0.222	0.11 (0, 0.3)
UDP-GlcNAc	332[a] ± 122	421[ab] ± 184	553[b] ± 194	**0.016**	**0.27 (0.04, 0.47)**
UMP	313 ± 137	466 ± 273	599 ± 302	0.055	0.2 (0.01, 0.4)
Uracil	101[a] ± 82	298[b] ± 171	291[b] ± 119	**<0.001**	**0.54 (0.3, 0.68)**
Uridine	158[a] ± 103	295[ab] ± 172	391[b] ± 213	**0.005**	**0.33 (0.08, 0.52)**
Valine	918 ± 303	852 ± 266	775 ± 165	0.475	0.06 (0, 0.22)

(1) Metabolite concentrations (mean ± SD) are expressed in nmol/g wet weight.

(2) The effect of treatment was assessed using ANOVA and FDR-correction applied based on the number of quantified metabolites in liver tissue. Statistical significance was defined as FDR-corrected $p < 0.05$ (denoted *p* in the table above) and are indicated in bolded text. When a significant effect of treatment was noted, Tukey HSD with FDR-correction was used for post hoc testing. Differing superscripts indicate significant differences between treatment groups. [+] indicates no significant differences between treatment groups were observed in post hoc testing.

(3) Effect size expressed as generalized eta-squared (η^2_G) with 95% confidence interval.

Abbreviations: GPC, glycerophosphocholine; O-PC, O-phosphocholine; UDP-Gal, UDP-galactose; UDP-Glc, UDP-glucose, UDP-GlcNAc, UDP-N-Acetylglucosamine

Table S3. Skeletal muscle metabolite concentrations in Con, ID, and ID+Abx piglets. [(1)]

**Metabolite**	**[Con]**	**[ID]**	**[ID+Abx]**	***p*** **[(2)]**	**η^2_G** **[(3)]**
2-Aminoadipate	224ª ± 111	176ª ± 112	60ᵇ ± 34	**<0.001**	**0.51 (0.27, 0.66)**
2-Aminobutyrate	37 ± 10	41 ± 10	49 ± 14	0.097	0.15 (0, 0.35)
Acetate	228 ± 114	264 ± 93	196 ± 42	0.382	0.06 (0, 0.23)
Acetone	14 ± 7	12 ± 4	14 ± 4	0.717	0.02 (0, 0.13)
Alanine	3131 ± 1142	4091 ± 852	3503 ± 840	0.084	0.16 (0, 0.36)
Asparagine	311 ± 163	354 ± 87	221 ± 104	0.162	0.12 (0, 0.31)
Aspartate	638 ± 261	498 ± 112	564 ± 326	0.450	0.05 (0, 0.21)
β-Alanine	1464ª ± 849	1121ᵃᵇ ± 693	677ᵇ ± 659	**0.015**	**0.28 (0.05, 0.47)**
Betaine	211ª ± 147	144ᵃᵇ ± 62	94ᵇ ± 86	**0.019**	**0.25 (0.03, 0.45)**
Carnitine	1060 ± 212	896 ± 139	1138 ± 575	0.410	0.06 (0, 0.22)
Creatine	26907 ± 3862	29783 ± 5414	31099 ± 7014	0.144	0.13 (0, 0.32)
Creatinine	133 ± 34	152 ± 41	118 ± 34	0.189	0.1 (0, 0.29)
Formate	96 ± 58	75 ± 52	73 ± 29	0.320	0.07 (0, 0.25)
Fumarate	57ª ± 23	47ᵃᵇ ± 9	33ᵇ ± 12	**0.012**	**0.29 (0.06, 0.49)**
Glc-1-P	186 ± 50	200 ± 47	235 ± 64	0.141	0.13 (0, 0.32)
Glucose	2052 ± 472	1981 ± 530	1808 ± 467	0.450	0.05 (0, 0.2)
Glutamate	4459 ± 1241	4919 ± 801	4820 ± 1200	0.450	0.05 (0, 0.2)
Glutamine	6994 ± 3778	11758 ± 2254	9016 ± 3800	**0.032⁺**	**0.22 (0.02, 0.42)**
Glutathione	561 ± 224	662 ± 118	699 ± 229	0.164	0.11 (0, 0.31)
Glycine	4167 ± 1730	3226 ± 1363	2187 ± 829	0.062	0.18 (0, 0.38)
GPC	694ª ± 301	706ª ± 308	1575ᵇ ± 898	**0.005**	**0.32 (0.08, 0.51)**
GTP	113ª ± 17	100ᵃᵇ ± 15	93ᵇ ± 12	**0.016**	**0.26 (0.04, 0.46)**
Inosine	152 ± 77	168 ± 56	134 ± 45	0.476	0.04 (0, 0.19)
Isoleucine	146 ± 27	148 ± 20	179 ± 87	0.424	0.05 (0, 0.22)
Lactate	20479ª ± 4600	19847ᵃᵇ ± 6030	14619ᵇ ± 2730	**0.016**	**0.26 (0.04, 0.46)**
Leucine	116ª ± 29	164ᵃᵇ ± 25	186ᵇ ± 63	**<0.001**	**0.44 (0.18, 0.61)**
Malate	892ª ± 307	738ª ± 131	469ᵇ ± 191	**0.001**	**0.4 (0.15, 0.58)**
myo-Inositol	747ª ± 477	759ª ± 365	2105ᵇ ± 1017	**<0.001**	**0.45 (0.2, 0.62)**
NAD	346 ± 50	396 ± 116	325 ± 63	0.189	0.1 (0, 0.29)
Niacinamide	127 ± 31	139 ± 27	183 ± 86	0.062	0.18 (0, 0.38)
O-Acetylcarnitine	294 ± 66	335 ± 94	322 ± 100	0.507	0.04 (0, 0.18)
Pantothenate	37 ± 16	46 ± 13	49 ± 14	0.097	0.15 (0, 0.34)
Phenylalanine	55 ± 26	89 ± 17	95 ± 30	**0.041⁺**	**0.21 (0.01, 0.41)**
Phosphocreatine	3066 ± 1897	1985 ± 1063	3531 ± 2037	0.283	0.08 (0, 0.26)
Propylene glycol	74 ± 50	37 ± 29	86 ± 38	**0.046⁺**	**0.2 (0.01, 0.4)**
Pyruvate	129 ± 47	104 ± 47	101 ± 36	0.189	0.11 (0, 0.29)
Succinate	379ᵃᵇ ± 328	551ª ± 202	218ᵇ ± 92	**0.004**	**0.34 (0.1, 0.53)**
Taurine	7152 ± 2643	6978 ± 1229	10367 ± 3637	0.054	0.19 (0, 0.39)
Threonine	397 ± 152	319 ± 51	506 ± 164	0.086	0.16 (0, 0.36)
Tyrosine	268 ± 78	286 ± 101	187 ± 74	**0.027⁺**	**0.23 (0.02, 0.43)**
Valine	434 ± 56	447 ± 85	411 ± 136	0.450	0.05 (0, 0.2)

[(1)] Metabolite concentrations (mean ± SD) are expressed in nmol/g wet weight.

[2] The effect of treatment was assessed using ANOVA and FDR-correction applied based on the number of quantified metabolites in skeletal muscle tissue. Statistical significance was defined as FDR-corrected $p < 0.05$ (denoted p in the table above) and are indicated in bolded text. When a significant effect of treatment was noted, Tukey HSD with FDR-correction was used for post hoc testing. Differing superscripts indicate significant differences between treatment groups. $^{+}$ indicates no significant differences between treatment groups were observed in post hoc testing.

[3] Effect size expressed as generalized eta-squared (η^2_G) with 95% confidence interval.

Abbreviations: Glc-1-P, glucose-1-phosphate; GPC, glycerophosphocholine

Table S4. Hippocampal metabolite concentrations in Con, ID, and ID+Abx piglets. [(1)]

Metabolite	[Con]	[ID]	[ID+Abx]	p [(2)]	η^2_G [(3)]
Acetate	850 ± 153	863 ± 129	758 ± 122	0.267	0.09 (0, 0.27)
Alanine	1807[ab] ± 285	2088[a] ± 311	1613[b] ± 189	**0.003**	**0.3 (0.06, 0.49)**
Ascorbate	371 ± 146	327 ± 101	319 ± 113	0.747	0.02 (0, 0.15)
Aspartate	2520[a] ± 228	2190[b] ± 167	2430[ab] ± 314	**0.008**	**0.27 (0.04, 0.47)**
Choline	181[a] ± 66	150[ab] ± 44	112[b] ± 23	**0.008**	**0.27 (0.05, 0.47)**
Creatine	9777[a] ± 657	8851[b] ± 653	9876[a] ± 712	**0.003**	**0.31 (0.07, 0.5)**
Dimethylglycine	26[a] ± 2	24[b] ± 2	26[ab] ± 2	**0.020**	**0.22 (0.02, 0.42)**
Formate	382 ± 66	424 ± 89	385 ± 78	0.406	0.06 (0, 0.23)
Fumarate	42 ± 7	39 ± 11	42 ± 7	0.591	0.04 (0, 0.19)
GABA	2879 ± 268	2844 ± 401	2568 ± 198	**0.043[+]**	**0.18 (0, 0.38)**
Glutamate	13980 ± 855	13838 ± 986	13589 ± 1020	0.662	0.03 (0, 0.17)
Glutamine	7399 ± 1272	8910 ± 925	7460 ± 1793	**0.033[+]**	**0.2 (0.01, 0.4)**
Glutarate	106 ± 20	107 ± 22	111 ± 18	0.820	0.01 (0, 0.12)
Glutathione	1068 ± 187	1070 ± 215	1010 ± 181	0.817	0.02 (0, 0.13)
Glycerol	645[a] ± 149	684[a] ± 208	431[b] ± 45	**0.001**	**0.42 (0.17, 0.6)**
Glycine	1595[a] ± 227	1975[b] ± 287	1489[a] ± 368	**0.003**	**0.31 (0.07, 0.5)**
GPC	2927[a] ± 335	2347[b] ± 451	2491[ab] ± 285	**0.001**	**0.38 (0.12, 0.56)**
Hypoxanthine	179[a] ± 64	198[a] ± 98	104[b] ± 19	**0.002**	**0.36 (0.11, 0.54)**
Inosine	469[a] ± 79	476[a] ± 96	349[b] ± 54	**0.002**	**0.35 (0.1, 0.54)**
Isoleucine	139 ± 31	128 ± 18	120 ± 13	0.195	0.11 (0, 0.29)
Lactate	22304[a] ± 2663	25077[b] ± 2633	24482[ab] ± 824	**0.010**	**0.26 (0.04, 0.46)**
Leucine	143[a] ± 19	171[b] ± 20	156[ab] ± 14	**0.003**	**0.3 (0.06, 0.49)**
myo-Inositol	10232[a] ± 956	8372[b] ± 759	9491[ab] ± 1609	**0.002**	**0.35 (0.11, 0.54)**
N-Acetylaspartate	9236 ± 838	9041 ± 720	8926 ± 640	0.686	0.03 (0, 0.17)
Niacinamide	139 ± 29	136 ± 13	136 ± 18	0.976	0 (0, 0)
O-PC	483 ± 89	484 ± 52	479 ± 62	0.976	0 (0, 0)
O-PE	2529 ± 244	2443 ± 198	2480 ± 179	0.686	0.03 (0, 0.16)
Pantothenate	29 ± 9	30 ± 5	24 ± 10	0.111	0.14 (0, 0.33)
Phenylalanine	67[a] ± 16	91[b] ± 15	72[ab] ± 14	**0.006**	**0.29 (0.06, 0.48)**
Propylene glycol	54 ± 28	51 ± 43	68 ± 34	0.260	0.09 (0, 0.27)
Serine	1778 ± 318	1685 ± 227	1460 ± 423	**0.039[+]**	**0.19 (0, 0.39)**
Succinate	446[a] ± 89	571[b] ± 95	393[a] ± 31	**0.001**	**0.4 (0.15, 0.58)**
Taurine	1700 ± 347	1894 ± 271	1699 ± 350	0.335	0.07 (0, 0.25)
Threonine	838[a] ± 250	586[b] ± 86	828[ab] ± 194	**0.014**	**0.24 (0.03, 0.44)**
Tyrosine	272[a] ± 69	248[a] ± 56	148[b] ± 46	**0.001**	**0.45 (0.2, 0.62)**
UDP-GlcNAc	97 ± 15	87 ± 16	97 ± 19	0.335	0.07 (0, 0.25)
UMP	99[ab] ± 20	87[a] ± 23	114[b] ± 8	**0.025**	**0.21 (0.01, 0.41)**
Uridine	157 ± 23	146 ± 27	135 ± 22	0.095	0.14 (0, 0.34)
Valine	378[a] ± 64	341[ab] ± 26	289[b] ± 33	**0.001**	**0.39 (0.13, 0.57)**

[(1)] Metabolite concentrations (mean ± SD) are expressed in nmol/g wet weight.

[(2)] The effect of treatment was assessed using ANOVA and FDR-correction applied based on the number of quantified metabolites in hippocampal tissue. Statistical significance was defined as

FDR-corrected $p < 0.05$ (denoted p in the table above) and are indicated in bolded text. When a significant effect of treatment was noted, Tukey HSD with FDR-correction was used for post hoc testing. Differing superscripts indicate significant differences between treatment groups. $^{+}$ indicates no significant differences between treatment groups were observed in post hoc testing.

(3) Effect size expressed as generalized eta-squared (η^2_G) with 95% confidence interval.

Abbreviations: GABA, γ-aminobutyric acid; GPC, glycerophosphocholine; O-PC, O-phosphocholine; O-PE, O-phosphoethanolamine; UDP-GlcNAc, UDP-N-Acetylglucosamine

Table S5. Kidney metabolite concentrations in Con* and Con*+Abx piglets.[1]

Metabolite	[Con*]	[Con*+Abx]	*p* [2]	Hedge's *g* [3]
2-Aminoadipate	1161 + 462	779 + 320	0.727	0.89 (-0.35, 2.14)
2-Aminobutyrate	63 + 34	41 + 16	0.727	0.79 (-0.44, 2.02)
2-Hydroxyisovalerate	47 + 41	42 + 41	0.765	0.25 (-0.94, 1.45)
3-Aminoisobutyrate	30 + 8	27 + 10	0.759	0.34 (-0.85, 1.54)
3-Hydroxybutyrate	40 + 15	35 + 10	0.759	0.29 (-0.91, 1.48)
Acetate	396 + 132	362 + 76	0.816	0.19 (-1.00, 1.38)
Acetone	66 + 26	74 + 27	0.759	0.29 (-0.90, 1.48)
ADP	360 + 95	310 + 66	0.727	0.57 (-0.64, 1.78)
Alanine	5111 + 1695	3648 + 377	0.727	1.11 (-0.17, 2.38)
AMP	1255 + 481	1046 + 189	0.748	0.45 (-0.75, 1.66)
Asparagine	915 + 306	754 + 174	0.727	0.56 (-0.65, 1.77)
Aspartate	3241 + 1087	2824 + 489	0.759	0.37 (-0.83, 1.57)
β-Alanine	485 + 264	396 + 92	0.854	0.15 (-1.04, 1.34)
Betaine	1947 + 1325	1128 + 659	0.727	0.65 (-0.57, 1.86)
Choline	849 + 172	867 + 183	0.903	0.08 (-1.11, 1.27)
Creatine	1106 + 523	1263 + 459	0.759	0.33 (-0.87, 1.52)
Cysteine	396 + 99	350 + 43	0.727	0.50 (-0.70, 1.71)
Formate	149 + 52	140 + 36	0.877	0.12 (-1.06, 1.31)
Fumarate	163 + 51	152 + 11	0.886	0.11 (-1.08, 1.30)
GABA	111 + 39	88 + 41	0.727	0.57 (-0.64, 1.79)
Glucose	3168 + 1091	2504 + 901	0.727	0.50 (-0.71, 1.70)
Glutamate	17149 + 5830	13229 + 2122	0.727	0.76 (-0.47, 1.99)
Glutamine	1970 + 776	1500 + 289	0.727	0.78 (-0.45, 2.01)
Glutathione	236 + 42	195 + 71	0.727	0.66 (-0.56, 1.88)
Glycerol	927 + 240	680 + 362	0.727	0.58 (-0.63, 1.80)
Glycine	14013 + 4556	10759 + 2115	0.727	0.81 (-0.43, 2.04)
GPC	3206 + 946	2517 + 1467	0.727	0.69 (-0.53, 1.92)
GTP	120 + 45	103 + 25	0.759	0.36 (-0.84, 1.56)
Guanosine	138 + 50	122 + 21	0.759	0.31 (-0.88, 1.51)
Hippurate	78 + 57	70 + 43	0.759	0.31 (-0.89, 1.50)
Hypoxanthine	1045 + 340	961 + 187	0.825	0.18 (-1.01, 1.37)
IMP	129 + 92	106 + 61	0.765	0.26 (-0.93, 1.46)
Inosine	741 + 229	625 + 100	0.727	0.56 (-0.65, 1.77)
Isoleucine	606 + 187	504 + 79	0.727	0.59 (-0.63, 1.80)
Lactate	14284 + 4208	11090 + 1874	0.727	0.86 (-0.38, 2.10)
Leucine	856 + 267	733 + 122	0.727	0.49 (-0.71, 1.70)
Lysine	970 + 264	835 + 132	0.727	0.55 (-0.66, 1.76)
Malate	2390 + 820	2067 + 235	0.759	0.36 (-0.84, 1.56)
Methionine	332 + 99	296 + 61	0.759	0.34 (-0.86, 1.54)
π-Methylhistidine	342 + 79	326 + 51	0.814	0.20 (-0.99, 1.40)
myo-inositol	15539 + 4516	11352 + 4085	0.727	1.00 (-0.26, 2.25)
NAD	654 + 245	512 + 166	0.727	0.63 (-0.59, 1.85)
NADP	72 + 26	57 + 16	0.727	0.61 (-0.61, 1.82)
Niacinamide	268 + 70	280 + 31	0.759	0.32 (-0.88, 1.51)
O-PC	1880 + 609	1690 + 424	0.759	0.28 (-0.91, 1.47)
O-PE	2832 + 403	2640 + 587	0.759	0.41 (-0.79, 1.61)
Phenylalanine	460 + 126	398 + 69	0.727	0.52 (-0.69, 1.73)

**Metabolite**	**[Con*]**	**[Con*+Abx]**	***p*** [(2)]	**Hedge's *g*** [(3)]
Propylene glycol	2138 + 1284	1777 + 1363	0.748	0.45 (-0.76, 1.65)
Serine	2524 + 762	1953 + 333	0.727	0.87 (-0.37, 2.11)
Succinate	795 + 316	658 + 206	0.748	0.45 (-0.76, 1.65)
Taurine	5055 + 2007	4987 + 1211	0.905	0.06 (-1.12, 1.25)
Threonine	1255 + 510	968 + 91	0.727	0.59 (-0.63, 1.80)
Tryptophan	114 + 28	114 + 19	0.903	0.08 (-1.11, 1.27)
Tyrosine	598 + 177	529 + 83	0.759	0.38 (-0.82, 1.58)
UDP-Gal	76 + 46	40 + 30	0.727	0.81 (-0.43, 2.04)
UDP-Glc	149 + 64	117 + 34	0.727	0.52 (-0.68, 1.73)
UDP-GlcNAc	177 + 64	145 + 25	0.727	0.58 (-0.63, 1.80)
UDP-Glucuronate	192 + 115	131 + 38	0.727	0.59 (-0.62, 1.81)
UMP	178 + 79	143 + 18	0.727	0.50 (-0.71, 1.70)
Uracil	157 + 68	137 + 37	0.765	0.25 (-0.95, 1.44)
Uridine	143 + 55	113 + 40	0.727	0.58 (-0.64, 1.79)
Valine	908 + 235	826 + 105	0.759	0.36 (-0.84, 1.56)

[(1)] Metabolite concentrations (mean ± SD) are expressed in nmol/g wet weight.

[(2)] The effect of antibiotic treatment on individual metabolite concentrations between control (Con*, n = 6) and antibiotic-treated (Con*+Abx, n = 6) piglets in Cohort 3 was assessed using ANOVA and FDR-correction applied based on the number of quantified metabolites in the kidney tissue. Statistical significance was defined as FDR-corrected $p < 0.05$ (denoted *p* in the table above).

[(3)] Effect size presented as Hedge's *g* with 95% confidence interval.

Abbreviations: GABA, γ-aminobutyric acid; GPC, glycerophosphocholine; O-PC, O-phosphocholine; O-PE, O-phosphoethanolamine; UDP-Gal, UDP-galactose; UDP-Glc, UDP-glucose, UDP-GlcNAc, UDP-N-Acetylglucosamine

Table S6. Liver metabolite concentrations in Con* and Con*+Abx piglets. [(1)]

Metabolite	[Con*]	[Con*+Abx]	p [(2)]	Hedge's g [(3)]
2-Aminoadipate	351 + 121	245 + 121	0.893	0.93 (-0.32, 2.18)
2-Aminobutyrate	56 + 33	42 + 22	0.922	0.52 (-0.69, 1.72)
2-Hydroxyisovalerate	14 + 5	9 + 9	0.893	0.83 (-0.40, 2.07)
3-Hydroxybutyrate	20 + 10	14 + 9	0.922	0.54 (-0.67, 1.75)
Acetate	296 + 207	290 + 143	0.979	0.09 (-1.10, 1.28)
Acetone	39 + 3	42 + 9	0.957	0.38 (-0.82, 1.58)
ADP	372 + 48	349 + 68	0.957	0.40 (-0.80, 1.60)
Alanine	5117 + 1660	3854 + 333	0.734	1.15 (-0.13, 2.44)
AMP	1071 + 213	935 + 201	0.893	0.60 (-0.62, 1.81)
Asparagine	816 + 156	790 + 95	0.979	0.13 (-1.06, 1.31)
Aspartate	3095 + 1089	2671 + 501	0.957	0.42 (-0.78, 1.62)
β-Alanine	420 + 399	262 + 45	0.957	0.43 (-0.77, 1.63)
Betaine	1159 + 1208	1252 + 705	0.957	0.34 (-0.86, 1.53)
Butyrate	19 + 2	17 + 9	0.922	0.54 (-0.67, 1.74)
Choline	350 + 114	385 + 103	0.957	0.35 (-0.84, 1.55)
Creatine	608 + 116	750 + 178	0.893	0.80 (-0.44, 2.03)
Cysteine	539 + 487	375 + 114	0.979	0.28 (-0.91, 1.48)
Cytidine	52 + 8	52 + 10	0.979	0.06 (-1.13, 1.25)
Formate	83 + 18	83 + 20	0.979	0.04 (-1.15, 1.22)
Fumarate	103 + 13	108 + 14	0.957	0.35 (-0.84, 1.55)
GPC	8691 + 1351	10216 + 1218	0.734	1.11 (-0.17, 2.38)
GTP	68 + 7	62 + 11	0.893	0.61 (-0.61, 1.82)
Glucose	28971 + 5430	27506 + 6498	0.979	0.27 (-0.92, 1.46)
Glutamate	5737 + 819	5053 + 1130	0.893	0.71 (-0.51, 1.94)
Glutamine	3558 + 459	2919 + 348	0.734	1.47 (0.13, 2.81)
Glutathione	1400 + 550	1336 + 328	0.992	0.02 (-1.17, 1.20)
Glycerol	660 + 158	622 + 118	0.979	0.23 (-0.96, 1.42)
Glycine	4789 + 997	4118 + 618	0.893	0.77 (-0.46, 2.00)
Guanosine	75 + 17	73 + 9	0.979	0.10 (-1.09, 1.29)
Hippurate	65 + 20	49 + 32	0.893	0.61 (-0.60, 1.83)
Hypoxanthine	90 + 46	91 + 23	0.979	0.21 (-0.98, 1.41)
Inosine	779 + 175	705 + 101	0.957	0.37 (-0.83, 1.57)
Isoleucine	390 + 87	377 + 61	0.979	0.10 (-1.09, 1.29)
Lactate	13382 + 1102	12063 + 1473	0.893	0.90 (-0.35, 2.15)
Leucine	800 + 115	726 + 72	0.893	0.65 (-0.57, 1.86)
Lysine	738 + 164	681 + 133	0.979	0.30 (-0.89, 1.49)
Malate	2747 + 595	2674 + 447	0.979	0.10 (-1.09, 1.29)
Mannose	600 + 119	605 + 108	0.979	0.06 (-1.13, 1.25)
Methionine	241 + 63	246 + 42	0.979	0.15 (-1.04, 1.34)
π-Methylhistidine	401 + 126	409 + 105	0.979	0.12 (-1.07, 1.31)
myo-inositol	1657 + 188	1644 + 272	0.979	0.09 (-1.09, 1.28)
NAD	421 + 65	338 + 57	0.734	1.27 (-0.03, 2.57)
NADP	172 + 19	147 + 24	0.734	1.10 (-0.17, 2.37)
O-PC	2871 + 1134	2858 + 1134	0.992	0.01 (-1.18, 1.19)
Niacinamide	409 + 83	395 + 82	0.979	0.12 (-1.07, 1.31)
Ornithine	482 + 151	513 + 147	0.979	0.21 (-0.98, 1.40)
Phenylalanine	292 + 50	291 + 28	0.979	0.04 (-1.14, 1.23)

Metabolite	[Con*]	[Con*+Abx]	p [(2)]	Hedge's g [(3)]
Proline	682 + 125	598 + 111	0.893	0.70 (-0.53, 1.92)
Propylene glycol	356 + 166	296 + 200	0.957	0.46 (-0.75, 1.66)
Serine	3875 + 1599	3680 + 932	0.979	0.04 (-1.15, 1.22)
Succinate	576 + 134	523 + 159	0.957	0.38 (-0.82, 1.58)
Taurine	1947 + 331	2146 + 849	0.979	0.14 (-1.05, 1.33)
Threonine	854 + 187	743 + 165	0.893	0.62 (-0.59, 1.84)
Tryptophan	66 + 20	71 + 11	0.957	0.35 (-0.85, 1.55)
Tyrosine	395 + 106	404 + 68	0.979	0.16 (-1.03, 1.35)
UDP-Gal	187 + 31	168 + 27	0.893	0.61 (-0.61, 1.82)
UDP-Glucose	593 + 155	510 + 120	0.922	0.51 (-0.69, 1.72)
UDP-GlcNAc	272 + 39	265 + 45	0.979	0.17 (-1.02, 1.36)
UMP	263 + 48	202 + 64	0.734	1.08 (-0.19, 2.35)
Uracil	75 + 28	75 + 34	0.979	0.08 (-1.10, 1.27)
Uridine	133 + 48	103 + 12	0.893	0.78 (-0.45, 2.01)
Valine	613 + 145	618 + 84	0.979	0.12 (-1.06, 1.31)

(1) Metabolite concentrations (mean ± SD) are expressed in nmol/g wet weight.

(2) The effect of antibiotic treatment on individual metabolite concentrations between control (Con*, n = 6) and antibiotic-treated (Con*+Abx, n = 6) piglets in Cohort 3 was assessed using ANOVA and FDR-correction applied based on the number of quantified metabolites in the liver tissue. Statistical significance was defined as FDR-corrected $p < 0.05$ (denoted p in the table above).

(3) Effect size presented as Hedge's g with 95% confidence interval.

Abbreviations: GPC, glycerophosphocholine; O-PC, O-phosphocholine; UDP-Gal, UDP-galactose; UDP-Glc, UDP-glucose, UDP-GlcNAc, UDP-N-Acetylglucosamine

Table S7. Skeletal muscle metabolite concentrations in Con* and Con*+Abx piglets. (1)

Metabolite	[Con*]	[Con*+Abx]	*p* (2)	Hedge's *g* (3)
2-Aminoadipate	150 + 82	119 + 74	0.901	0.47 (-0.73, 1.68)
2-Aminobutyrate	40 + 17	32 + 12	0.901	0.45 (-0.75, 1.65)
Acetate	244 + 120	320 + 197	0.901	0.30 (-0.90, 1.49)
Acetone	14 + 7	17 + 10	0.910	0.18 (-1.01, 1.37)
Alanine	2902 + 1101	2119 + 601	0.901	0.87 (-0.37, 2.12)
Asparagine	158 + 110	126 + 42	0.901	0.25 (-0.94, 1.45)
Aspartate	706 + 245	460 + 186	0.901	1.10 (-0.18, 2.37)
β-Alanine	1476 + 1459	863 + 481	0.901	0.53 (-0.68, 1.74)
Betaine	257 + 101	286 + 168	0.971	0.06 (-1.13, 1.25)
Carnitine	1142 + 111	1075 + 201	0.901	0.45 (-0.75, 1.65)
Creatine	28793 + 1965	28626 + 2148	0.971	0.08 (-1.11, 1.27)
Creatinine	135 + 46	154 + 24	0.901	0.58 (-0.63, 1.79)
Formate	89 + 72	112 + 82	0.901	0.26 (-0.93, 1.45)
Fumarate	32 + 8	33 + 11	0.971	0.02 (-1.17, 1.21)
Glc-1-P	199 + 40	216 + 63	0.901	0.23 (-0.97, 1.42)
Glucose	1645 + 324	1897 + 355	0.901	0.71 (-0.51, 1.94)
Glutamate	5617 + 1532	3911 + 1377	0.901	1.09 (-0.18, 2.36)
Glutamine	3999 + 1170	5435 + 2580	0.901	0.53 (-0.68, 1.74)
Glutathione	385 + 158	421 + 68	0.901	0.41 (-0.79, 1.61)
Glycine	5969 + 2460	5149 + 1457	0.901	0.26 (-0.93, 1.45)
GPC	364 + 133	453 + 242	0.901	0.37 (-0.83, 1.57)
GTP	97 + 15	97 + 12	0.971	0.03 (-1.16, 1.22)
Inosine	76 + 17	71 + 12	0.901	0.25 (-0.94, 1.45)
Isoleucine	125 + 28	114 + 27	0.901	0.41 (-0.79, 1.61)
Lactate	16755 + 4542	17755 + 2467	0.901	0.36 (-0.84, 1.55)
Leucine	106 + 18	105 + 18	0.971	0.07 (-1.12, 1.25)
Malate	523 + 179	481 + 165	0.901	0.27 (-0.92, 1.46)
myo-inositol	997 + 203	1098 + 353	0.901	0.25 (-0.94, 1.44)
NAD	417 + 70	386 + 58	0.901	0.45 (-0.75, 1.66)
Niacinamide	118 + 39	96 + 24	0.901	0.59 (-0.62, 1.81)
O-Acetylcarnitine	297 + 46	287 + 42	0.910	0.19 (-1.00, 1.38)
Pantothenate	62 + 13	75 + 19	0.901	0.66 (-0.56, 1.88)
Phenylalanine	56 + 18	59 + 16	0.910	0.17 (-1.02, 1.36)
Phosphocreatine	6206 + 2015	5283 + 1041	0.901	0.44 (-0.76, 1.64)
Propylene glycol	253 + 191	316 + 177	0.901	0.23 (-0.96, 1.42)
Pyruvate	155 + 56	196 + 45	0.901	0.85 (-0.39, 2.09)
Succinate	205 + 33	181 + 60	0.901	0.54 (-0.67, 1.75)
Taurine	6019 + 1183	6308 + 1971	0.971	0.07 (-1.12, 1.26)
Threonine	408 + 230	354 + 65	0.971	0.04 (-1.15, 1.23)
Tyrosine	76 + 15	91 + 18	0.901	0.85 (-0.39, 2.09)
Valine	120 + 69	143 + 41	0.901	0.53 (-0.68, 1.74)

(1) Metabolite concentrations (mean ± SD) are expressed in nmol/g wet weight.

(2) The effect of antibiotic treatment on individual metabolite concentrations between control (Con*, n = 6) and antibiotic-treated (Con*+Abx, n = 6) piglets in Cohort 3 was assessed using

ANOVA and FDR-correction applied based on the number of quantified metabolites in the skeletal muscle tissue. Statistical significance was defined as FDR-corrected $p < 0.05$ (denoted p in the table above).

(3) Effect size presented as Hedge's g with 95% confidence interval.

Abbreviations: Glc-1-P, glucose-1-phosphate; GPC, glycerophosphocholine

Table S8. Hippocampal metabolite concentrations in Con* and Con*+Abx piglets.(1)

Metabolite	[Con*]	[Con*+Abx]	*p* (2)	Hedge's *g* (3)
Acetate	448 + 173	433 + 128	0.983	0.01 (-1.30, 1.33)
Alanine	1827 + 165	1557 + 165	0.113	1.48 (0, 2.97)
Ascorbate	242 + 38	215 + 53	0.452	0.57 (-0.77, 1.91)
Aspartate	2758 + 295	2517 + 336	0.374	0.69 (-0.66, 2.05)
Choline	195 + 43	196 + 33	0.958	0.05 (-1.27, 1.37)
Creatine	12185 + 856	10808 + 776	0.113	1.53 (0.03, 3.03)
Dimethylglycine	33 + 1	30 + 3	0.120	1.43 (-0.05, 2.91)
Formate	337 + 19	317 + 13	0.164	1.14 (-0.28, 2.56)
Fumarate	49 + 4	43 + 3	0.113	1.60 (0.09, 3.12)
GABA	3207 + 263	2838 + 185	0.113	1.49 (0, 2.98)
Glutamate	15334 + 1187	13091 + 1468	0.113	1.52 (0.03, 3.02)
Glutamine	8699 + 1180	8719 + 471	0.948	0.07 (-1.24, 1.39)
Glutarate	117 + 15	119 + 13	0.871	0.15 (-1.17, 1.47)
Glutathione	998 + 96	945 + 56	0.452	0.57 (-0.77, 1.92)
Glycerol	686 + 58	621 + 42	0.164	1.14 (-0.28, 2.57)
Glycine	1790 + 219	1533 + 151	0.138	1.29 (-0.16, 2.73)
GPC	3266 + 363	2853 + 339	0.176	1.05 (-0.36, 2.45)
Hypoxanthine	169 + 14	161 + 17	0.515	0.50 (-0.84, 1.83)
Inosine	514 + 69	418 + 32	0.113	1.67 (0.14, 3.19)
Isoleucine	144 + 26	120 + 14	0.176	1.07 (-0.34, 2.48)
Lactate	23596 + 2036	20200 + 1992	0.113	1.53 (0.03, 3.03)
Leucine	170 + 15	156 + 17	0.286	0.85 (-0.53, 2.22)
myo-inositol	13058 + 684	11317 + 1514	0.120	1.40 (-0.07, 2.87)
N-Acetylaspartate	10405 + 827	9309 + 391	0.113	1.55 (0.05, 3.06)
Niacinamide	179 + 14	156 + 14	0.113	1.50 (0.01, 2.99)
O-PC	599 + 119	466 + 45	0.138	1.32 (-0.14, 2.77)
O-PE	2744 + 448	2197 + 360	0.138	1.25 (-0.19, 2.69)
Pantothenate	67 + 9	52 + 9	0.113	1.49 (0, 2.98)
Phenylalanine	85 + 11	81 + 11	0.605	0.39 (-0.94, 1.72)
Propylene glycol	528 + 192	376 + 194	0.374	0.72 (-0.64, 2.08)
Serine	1851 + 393	1596 + 251	0.374	0.70 (-0.65, 2.06)
Succinate	475 + 44	413 + 32	0.113	1.48 (0, 2.97)
Taurine	1482 + 69	1455 + 149	0.769	0.25 (-1.07, 1.58)
Threonine	1133 + 319	756 + 225	0.138	1.26 (-0.18, 2.71)
Tyrosine	114 + 19	119 + 19	0.774	0.23 (-1.09, 1.55)
UDP-GlcNAc	98 + 11	89 + 15	0.452	0.59 (-0.76, 1.94)
UMP	108 + 15	93 + 11	0.176	1.06 (-0.35, 2.47)
Uridine	176 + 8	155 + 21	0.176	1.09 (-0.32, 2.51)
Valine	146 + 58	164 + 36	0.574	0.43 (-0.90, 1.77)

(1) Metabolite concentrations (mean ± SD) are expressed in nmol/g wet weight.

(2) The effect of antibiotic treatment on individual metabolite concentrations between control (Con*, n = 5) and antibiotic-treated (Con*+Abx, n = 5) piglets in Cohort 3 was assessed using ANOVA and FDR-correction applied based on the number of quantified metabolites in the

hippocampal tissue. Statistical significance was defined as FDR-corrected $p < 0.05$ (denoted p in the table above).

(3) Effect size presented as Hedge's g with 95% confidence interval.

Abbreviations: GABA, γ-aminobutyric acid; GPC, glycerophosphocholine; O-PC, O-phosphocholine; O-PE, O-phosphoethanolamine; UDP-GlcNAc, UDP-N-Acetylglucosamine
